# Implementation outcome instruments for use in physical healthcare settings: a systematic review

**DOI:** 10.1186/s13012-020-01027-6

**Published:** 2020-08-18

**Authors:** Zarnie Khadjesari, Sabah Boufkhed, Silia Vitoratou, Laura Schatte, Alexandra Ziemann, Christina Daskalopoulou, Eleonora Uglik-Marucha, Nick Sevdalis, Louise Hull

**Affiliations:** 1 Centre for Implementation Science, Health Service and Population Research Department, Institute of Psychiatry, Psychology and Neuroscience, King’s College London, 16 De Crespigny Park, London, SE5 8AF UK; 2 Behavioural and Implementation Science research group, School of Health Sciences, University of East Anglia, Edith Cavell Building, Norwich Research Park, Norwich, NR4 7TJ UK; 3 Psychometrics and Measurement Lab, Biostatistics and Health Informatics Department, Institute of Psychiatry, Psychology and Neuroscience, King’s College London, 16 De Crespigny Park, London, SE5 8AF UK; 4 Centre for Healthcare Innovation Research, City, University of London, Northampton Square, London, EC1V 0HB UK; 5 Health Service and Population Research Department, Institute of Psychiatry, Psychology and Neuroscience, King’s College London, 16 De Crespigny Park, London, SE5 8AF UK

**Keywords:** Implementation outcomes, Implementation science, Measurement properties, Psychometric properties, Systematic review

## Abstract

**Background:**

Implementation research aims to facilitate the timely and routine implementation and sustainment of evidence-based interventions and services. A glaring gap in this endeavour is the capability of researchers, healthcare practitioners and managers to quantitatively evaluate implementation efforts using psychometrically sound instruments. To encourage and support the use of precise and accurate implementation outcome measures, this systematic review aimed to identify and appraise studies that assess the measurement properties of quantitative implementation outcome instruments used in physical healthcare settings.

**Method:**

The following data sources were searched from inception to March 2019, with no language restrictions: MEDLINE, EMBASE, PsycINFO, HMIC, CINAHL and the Cochrane library. Studies that evaluated the measurement properties of implementation outcome instruments in physical healthcare settings were eligible for inclusion. Proctor et al.’s taxonomy of implementation outcomes was used to guide the inclusion of implementation outcomes: acceptability, appropriateness, feasibility, adoption, penetration, implementation cost and sustainability. Methodological quality of the included studies was assessed using the COnsensus-based Standards for the selection of health Measurement INstruments (COSMIN) checklist. Psychometric quality of the included instruments was assessed using the Contemporary Psychometrics checklist (ConPsy). Usability was determined by number of items per instrument.

**Results:**

Fifty-eight publications reporting on the measurement properties of 55 implementation outcome instruments (65 scales) were identified. The majority of instruments assessed acceptability (*n* = 33), followed by appropriateness (*n* = 7), adoption (*n* = 4), feasibility (*n* = 4), penetration (*n* = 4) and sustainability (*n* = 3) of evidence-based practice. The methodological quality of individual scales was low, with few studies rated as ‘excellent’ for reliability (6/62) and validity (7/63), and both studies that assessed responsiveness rated as ‘poor’ (2/2). The psychometric quality of the scales was also low, with 12/65 scales scoring 7 or more out of 22, indicating greater psychometric strength. Six scales (6/65) rated as ‘excellent’ for usability.

**Conclusion:**

Investigators assessing implementation outcomes quantitatively should select instruments based on their methodological and psychometric quality to promote consistent and comparable implementation evaluations. Rather than developing ad hoc instruments, we encourage further psychometric testing of instruments with promising methodological and psychometric evidence.

**Systematic review registration:**

PROSPERO 2017 CRD42017065348

Contributions to the literature

- This systematic review provides a repository of implementation outcome instruments that could be used in physical healthcare settings.
- It supports informed decision-making when selecting an instrument to measure implementation of evidence-based practice, by providing a benchmark of psychometric quality.
- It identifies key gaps in the measurement of core implementation outcomes that can be used to focus future research efforts.

## Introduction

Implementation research aims to close the research-to-practice gap, support scale-up of evidence-based interventions and reduce research waste [1, 2]. The field of implementation science has gained recognition over the last 10 years, with advances in effectiveness-implementation hybrid designs [3], frameworks that inform the determinants, processes and evaluation of implementation efforts [4–7], reporting guidance [8] and educational resources [9]. An essential component of these recent developments, and of the field as a whole, is the use of valid and reliable implementation outcome instruments. The widespread use of valid and pragmatic measures is needed to enable sophisticated statistical exploration of the complex associations between implementation effectiveness and factors thought to influence implementation success, implementation strategies and clinical effectiveness of evidence-based interventions [10].

Yet a glaring gap, albeit common in new specialities, remains in the capability of researchers, healthcare practitioners and managers to quantitatively evaluate implementation efforts using psychometrically sound measures [11]. To advance the science of implementation, a decade ago Proctor et al. proposed a working taxonomy of eight implementation outcomes, which are distinct from patient outcomes (e.g. symptoms, behaviours) and health service outcomes (e.g. efficiency, safety). Implementation outcomes are defined as ‘the effects of deliberate and purposive actions to implement new treatments, practices, and services’ [10]. This core set of implementation outcomes consists of the following: acceptability, appropriateness, adoption, feasibility, fidelity, implementation cost, penetration and sustainability of evidence-based practice. Despite Proctor et al.’s taxonomy, implementation scientists have highlighted slow progress towards widespread use of valid implementation outcome instruments, and that implementation research still focuses primarily on evaluating intervention effectiveness rather than implementation effectiveness [12]. This limits our understanding of factors affecting successful implementation.

Notable efforts to identify robust implementation outcome instruments at scale include systematic reviews of quantitative instruments validated in mental health settings [13] and public health and community settings [14], and more recently, a systematic scoping review has identified implementation outcomes and indicator-based implementation measures [15]. The vast majority of implementation outcome instruments are developed to assess the implementation of a specific intervention; therefore, a review of implementation outcome instruments validated in physical healthcare settings will retrieve a different set of instruments to those validated in mental health or community settings. Further, generic implementation outcome instruments need to be validated when applied in different settings before they can be used with confidence. The reviews in mental health and community settings reach similar conclusions; significant gaps in implementation outcome instrumentation exist and the limited number of instruments that do exist mostly lack psychometric strength.

There have been efforts to facilitate access to implementation outcome instruments, such as the development of online repositories. For example, the Society for Implementation Research Collaboration (SIRC) Implementation Outcomes Repository includes instruments that were validated in mental health settings [16]. These efforts represent significant advances in facilitating access to psychometrically sound and pragmatic quantitative implementation outcome instruments. To date, no attempt has been made to systematically identify and methodologically appraise quantitative implementation outcome instruments relevant to physical health settings. We aim to address this gap.

The aim of this systematic review is to identify and appraise studies that assess the measurement properties of quantitative implementation outcome instruments used in physical healthcare settings, to advance the use of precise and accurate measures.

## Methods

The protocol for this systematic review is published [17] and registered on the International Prospective Register of Systematic Reviews (PROSPERO) 2017 CRD42017065348. We followed the Preferred Reporting Items for Systematic Reviews and Meta-Analyses (PRISMA) statement [18]. In addition, we used the COnsensus-based Standards for the selection of health Measurement INstruments (COSMIN) guidance for systematic reviews of patient-reported outcome measures [19]. Whilst we are including implementation outcomes (assessed by different stakeholder groups) rather than health outcomes (assessed by patients alone), it is the self-report nature of the instruments and the inclusion of psychometric studies that make COSMIN reporting guidance applicable.

### Protocol deviations

The following bibliographic databases were listed in our protocol, but were not searched due to the vast literature identified by the six databases listed in the following paragraph and the limited capacity of our team: System for Information on Grey Literature in Europe (OpenGrey), ProQuest for theses, Web of Science Conference Proceedings Citation Index-Science (Thomson), Web of Science, Science Citation Index (Clarivate) for forward and backward citation tracking of included studies.

### Search strategy and selection criteria

The following databases were searched from inception to March 22, 2019, with no restriction on language: MEDLINE, EMBASE, PsycINFO and HMIC via the Ovid interface; CINAHL via the EBSCO Host interface; and the Cochrane library. Three sets of search terms were combined, using Boolean operators, to identify studies that evaluated the measurement properties of instruments that measure implementation outcomes. They describe the following: (1) the population/field of interest (i.e. implementation literature), (2) the implementation outcomes included in Proctor et al.’s taxonomy and their synonyms and (3) the measurement properties of instruments (e.g. test-retest reliability). The search terms were chosen after discussion with our stakeholder group and information specialist, and by browsing keywords and index terms (e.g. MeSH) assigned to relevant reviews (see published protocol for search terms [17]).

Studies that evaluated the measurement properties of instruments assessing an implementation outcome were eligible for inclusion. We applied Proctor et al.’s definitions of implementation outcomes to assess the eligibility of instruments, although constructs did not always fit neatly into the defined outcomes. Where the description of constructs fitted more than one of Proctor et al.’s implementation outcomes (e.g. acceptability and feasibility), the instrument was classified according to the predominant outcome at item level, determined through a detailed analysis and count of each instrument item (e.g. if an instrument contained 10 items assessing acceptability and two items assessing feasibility, the instruments would be categorised as an acceptability instrument). Where instruments measured additional constructs outside of Proctor et al.’s taxonomy, we classified according to the predominant eligible implementation outcome assessed. Where a predominant outcome was not obvious, we used the author’s own description of the instrument.

We included instruments that measured implementation of an evidence-based intervention or service in physical healthcare settings and excluded those in mental health, public health and community settings, as they have previously been identified in other systematic reviews [13, 14]. Instruments that assessed fidelity were excluded, as these are typically intervention specific and hence cannot be generalised [13].

### Study selection and data extraction

References identified from the search were imported into reference management software (Endnote V8). Duplicates were removed, and title and abstracts were screened independently by two reviewers (ZK 100%, LS 37.5%, AZ 37.5%, SB 25%). Potentially eligible studies were assessed in full text, independently by two reviewers (ZK and SB). Disagreements were discussed and resolved with senior team members (LH and NS). The following data were extracted using a pilot-tested data extraction form in Excel: (1) study characteristics: authors and year of publication, country, name of instrument and version, implementation outcome and level of analysis (i.e. organisation, provider, consumer); (2) methodological quality; (3) psychometric quality and (4) usability (i.e. number of items). Data extraction was performed by two postgraduate students in psychometrics (CD and EUM) and checked for accuracy (SV). Disagreements were resolved through discussion with the senior psychometrician (SV).

### Methodological quality

The COnsensus-based Standards for the selection of health Measurement INstruments (COSMIN) checklist was used to assess the methodological quality of the included studies [20]. The COSMIN checklist is a global measure of methodological quality, with separate criteria for nine different measurement properties [20]: reliability (internal consistency, test-retest reliability and, if applicable, inter-rater reliability), validity (content: face validity; criterion: predictive and concurrent validity; construct: convergent and discriminant validity) and dimensionality via the appropriate latent trait models (e.g. factor analysis, item response theory, item factor analysis). Each measurement property is assessed with 5 to 18 items evaluating the methodological quality of the study, each rated using a 4-point scale: ‘excellent’, ‘good’, ‘fair’ or ‘poor’. The global score for each measurement category (validity, reliability, responsiveness) is obtained by selecting the lowest rating of any item property (for details on the scoring process, see [20]). Definitions of reliability, validity and responsiveness are provided below:

- ‘Reliability is the degree to which a score or other measure remains unchanged upon test and retest (when no change is expected), or across different interviewers or assessors.
- Validity is the degree to which a measure assesses what it is intended to measure.
- Responsiveness represents the ability of a measure to detect change in an individual over time.’ [21] p. 316.

### Instrument/psychometric quality

Whilst the COSMIN checklist assesses the methodological quality of psychometric studies, it does not provide advice on the accuracy of the tests and resulting indices used to validate the instrument. To evaluate the psychometric quality of the included instruments, the Contemporary Psychometrics checklist (ConPsy) was developed for the purposes of this review by SV at the Psychometrics and Measurement Lab, Institute of Psychiatry, Psychology and Neuroscience, King’s College London [22]. Based on a literature review of seminal papers in the field of contemporary psychometrics and popularity of methods, the ConPsy checklist represents a consolidation of the most up-to-date statistical tools that complement the recommendations included within the COSMIN checklist. The ConPsy checklist will be updated every 2 years so that it remains contemporary. The psychometric strength of the ConPsy checklist is currently being evaluated as part of a separate study. To ensure the quality and accuracy of the psychometric data extraction in this review, as stated above, data extraction was performed by two postgraduate students in psychometrics (CD and EUM) and checked for accuracy by SV. The psychometric evaluation of ConPsy will explore the reliability (test-retest and inter-rater) and the convergent validity of the checklist. ConPsy assigns a maximum reliability score = 5, a maximum validity score = 5 and a maximum factor analysis score = 12. These are combined to provide a global score (minimum score = 0, maximum score = 22; higher scores indicate better psychometric quality). Further details of the ConPsy scoring system can be found in Additional file 1.

### Instrument usability

We assessed usability as number of items in an instrument and categorised in-line with previously developed usability criteria [13]: excellent, < 10 items; good, 10–49 items; adequate, 50–99 items and minimal, > 100 items. We explored the relationship between usability and both methodological and psychometric quality (as specified in our published protocol [17]). A Spearman’s correlation was used to assess the relationship between (1) usability and COSMIN reliability, (2) usability and COSMIN validity and (3) usability and ConPsy scores, where usability was treated as a categorical variable. It was not possible to assess the relationship between usability and responsiveness as only two studies investigated the latter. Where reliability and/or validity were not assessed or were unable to score (see Table 1), this was treated as missing data. Analyses were performed in SPSS v25.

**Table 1:** Summary of methodological quality, psychometric quality and usability, ranked by global COSMIN reliability score across implementation outcome

Reference	Implementation outcome Name of measurement instrument or instrument description	COSMIN			Usability* (number of items)	ConPsy score (/22)
		Reliability	Validity	Responsiveness		
	**Acceptability (number of instruments = 33):** the perception among implementation stakeholders that a given treatment, service, practice, or innovation is agreeable, palatable or satisfactory. Commonly used terms: satisfaction with various aspects of the innovation (e.g. content, complexity, comfort, delivery and credibility)					
Shaw et al. [23]	The Mind the Gap Scale—adolescent version	Excellent	Excellent	Not assessed	Good (22)	7
	The Mind the Gap Scale—parent version	Excellent	Excellent	Not assessed	Good (27)	7
Dow et al. [24]	The Person-Centred Health Care for Older Adults (PCHCOA) Survey	Excellent	Excellent	Not assessed	Good (31)	3
Dykes et al. [25]	The Impact of Health Information Technology (I-HIT) Scale	Excellent	Good	Not assessed	Good (29)	6
Brehaut et al. [26]	Ottawa acceptability of decision rules instrument (OADRI)	Excellent	Poor	Not assessed	Good (12)	3
Tomotaki et al. [27]	Evidence-Based Practice Questionnaire (EBPQ-J)—Japanese version	Good	Fair	Not assessed	Good (18)	4
Upton et al. [28]	Evidence-Based Practice Questionnaire (EBPQ)	Fair	Fair	Not assessed	Good (24)	7
Bhor et Mason [29]	A scale to assess attitudes of healthcare administrators towards the use of e-mail communication between patients and physicians	Fair	Fair	Not assessed	Good (20)	4
Phansalkar et al. [30]	Instrument for assessing clinicians’ perceptions about use of computerised protocols	Fair	Fair	Not assessed	Good (35)	3
Oliveira et al. [31]	CARDIOSATIS-Team scale	Fair	Poor	Not assessed	Good (11)	6
Wu et al. [32]	Healthcare professionals’ intention to use an adverse event reporting system	Fair	Poor	Not assessed	Good (17)	6
Melas et al. [33]	The Evidence-Based Practice Attitude Scale (EBPAS)—Greek version	Fair	Poor	Not assessed	Good (15)	5
Brouwers et al. [34]	Practitioner Feedback Questionnaire	Fair	Poor	Not assessed	Good (18)	4
Baker et al. [35]	The Attitudes Related to Trauma-Informed Care (ARTIC-45)	Fair	Poor	Not assessed	Good (45)	4
	The Attitudes Related to Trauma-Informed Care (ARTIC-35) Scale	Fair	Poor	Not assessed	Good (35)	4
	The Attitudes Related to Trauma-Informed Care (ARTIC-10) Scale—short version	Poor	Poor	Not assessed	Good (10)	2
Vanneste et al. [36]	A survey measuring acceptance of BelRAI, a web-based system enabling person-centred recording and data sharing across care settings	Fair	Poor	Not assessed	Good (31)	2
Bakas et al. [37]	A rating form measuring the satisfaction of the Telephone Assessment and Skill-Building Kit (TASK) intervention	Poor	Excellent	Not assessed	Excellent (9)	3
McConnell et al. [38]	Diffusion of Innovation in Long-Term Care (DOI-LTC) measurement battery-version for certified nursing assistants	Poor	Excellent	Not assessed	Good (40)	2
	Diffusion of Innovation in Long-Term Care (DOI-LTC) measurement battery-version for licensed nurses	Poor	Excellent	Not assessed	Adequate (50)	2
Atkinson [39]	A Questionnaire to Measure Perceived Attributes of eHealth Innovations	Poor	Good	Not assessed	Good (24)	4
Gagnon et al. [40]	A questionnaire based on the Technology Acceptance Model (TAM)	Poor	Fair	Not assessed	Good (33)	3
Ferrando et al. [41]	A questionnaire to measure convenience and satisfaction with a new internet-based tool for oral anticoagulation therapy telecontrol	Poor	Fair	Not assessed	Good (10)	2
Wilkinson et al. [42]	A survey measuring attitudes towards biomedical HIV prevention	Poor	Fair	Not assessed	Good (14)	2
Adu et al. [43]	A questionnaire measuring pharmacists and physician’s attitudes to antibiotic policies	Poor	Poor	Poor	Good (12)	2
Abetz et al. [44]	Cancer Therapy Satisfaction Questionnaire (CTSQ)	Poor	Poor	Poor	Good (21)	2
Blumenthal et al. [45]	Physiotherapy Mobile Acceptance Questionnaire (PTMAQ)	Poor	Poor	Not assessed	Good (30)	9
Weiner et al. [46]**	Acceptability of Intervention Measure (AIM)	Poor	Poor	Not assessed	Excellent (4)	8
Unni et al. [47]	A survey measuring satisfaction with electronic health records	Poor	Poor	Not assessed	Good (21)	6
Aggelidis et al. [48]	End user computing satisfaction (EUCS) survey	Poor	Poor	Not assessed	Good (49)	6
El-Den et al. [49]	Perinatal Depression (PND) Attitudes and Screening Acceptability Questionnaire (PASAQ)	Poor	Poor	Not assessed	Good (24)	5
Kramer et al. [50]	A generic questionnaire to detect physicians’ willingness to implement complex medical interventions	Poor	Poor	Not assessed	Good (41)	5
Frandes et al. [51]	An instrument assessing mobile technology acceptability in diabetes self-management	Poor	Poor	Not assessed	Good (29)	4
Rasoulzadeh et al. [52]	A questionnaire measuring acceptance of creating a nurses’ health monitoring system	Poor	Poor	Not assessed	Good (12)	3
Sockolow et al. [53]	Electronic Health Record Nurse Satisfaction (EHRNS) survey	Poor	Poor	Not assessed	Good (21)	3
Johnston et al. [54]	A questionnaire assessing physicians’ attitudes towards the computerisation of clinical practice	Poor	Poor	Not assessed	Good (20)	2
Bernhardsson et al. [55]	Evidence-Based Practice (EBP) Questionnaire—Swedish version	Poor	Poor	Not assessed	Good (31)	2
Yildiz et al. [56]	Evidence-Based Practice Attitude Scale (EBPAS-50)—Turkish version	Poor	Poor	Not assessed	Adequate (50)	0
Bevier et al. [57]	Questionnaire of three scoring items for current treatment satisfaction and factors of both clinical trial participation motivations and technology acceptance model	Poor	Not assessed	Not assessed	Good (34)	0
Wolf et al. [58]	Evidence-Based Practice Attitude Scale (EBPAS)	Not assessed	Excellent	Not assessed	Good (15)	5
Steed et al. [59]	Acceptability of Continuous Glucose Monitoring Devices (ACGMD) questionnaire	Not assessed	Fair	Not assessed	Adequate (64)	2
	**Appropriateness (number of instruments = 7):** the perceived fit, relevance or compatibility of the innovation or evidence-based practice for a given practice setting, provider or consumer and/or perceived fit of the innovation to address a particular issue or problem. Commonly used terms: perceived fit, relevance, compatibility, suitability, usefulness, and practicability					
Diego et al. [60]	A questionnaire to measure the attitude of anesthesiologists and residents regarding the use of the checklist in the perioperative period	Fair	Good	Not assessed	Excellent (7)	2
Park et al. [61]	A questionnaire measuring motivational factors for using wearable healthcare devices	Fair	Fair	Not assessed	Good (28)	8
Razmak et al. [62]	A techno-humanist model for e-health adoption of innovative technology	Fair	Fair	Not assessed	Good (32)	1
Joice et al. [63]	Perceived usefulness of a stroke workbook-based intervention measure	Poor	Fair	Not assessed	Good (15)	6
Xiao et al. [64]	Baylor EHR UX survey	Poor	Fair	Not assessed	Good (29)	3
Weiner et al. [46]**	Intervention Appropriateness Measure (IAM)	Poor	Poor	Not assessed	Excellent (4)	8
King et al. [65]	The Portal Survey on Satisfaction and Impact on Care	Poor	Poor	Not assessed	Good (38)	5
	**Adoption (number of instruments = 4):** the intention, initial decision or action to try or employ an innovation or evidence-based practice. Commonly used terms: uptake, utilisation, initial implementation and intention to try					
Nydegger et al. [66]	Strength of Implementation Intentions Scale (SIIS) for condom use	Fair	Fair	Not assessed	Good (22)	4
Everson et al. [67]	American Hospital Association IT (AHA-IT) Supplement Survey	Fair	Poor	Not assessed	Good (28)	6
Malo et al. [68]	A questionnaire evaluating nurses’ intention to use an electronic medical charting system	Poor	Poor	Not assessed	Good (46)	3
Kaltenbrunner et al. [69]	Lean in Healthcare Questionnaire (LiHcQ)	Unable to score***	Fair	Not assessed	Good (16)	7
	**Feasibility (number of instruments = 4):** the extent to which a new treatment or an innovation can be successfully used or carried out within a given agency or setting. Commonly used terms: actual fit or utility, suitability for everyday use and practicability					
Garcia-Smith et al. [70]	Instrument to test the Clinical Information Systems Success Model (CISSM)	Excellent	Poor	Not assessed	Good (26)	2
Schnall et al. [71]	Technology Acceptance Survey	Fair	Poor	Not assessed	Excellent (8)	3
Windsor et al. (2013)	The Smoking Cessation and Reduction in Pregnancy Treatment (SCRIPT) Adoption Scale	Poor	Poor	Not assessed	Good (28)	4
Weiner et al. [46]**	Feasibility of Intervention Measure (FIM)	Poor	Poor	Not assessed	Excellent (4)	8
	**Penetration (number of instruments = 4):** the integration of a practice within a service setting and its subsystems. Commonly used terms: level of institutionalisation, spread and service access					
Grooten et al. [72]	The Scaling Integrated Care in Context (SCIROCCO) tool	Good	Good	Not assessed	Good (12)	5
Slaghuis et al. [73]	A measurement instrument for spread of quality improvement in healthcare	Fair	Poor	Not assessed	Good (18)	4
Flanagan et al. [74]	The Prevention and Control of Antimicrobial resistance (PACAR) scale	Poor	Fair	Not assessed	Good (16)	6
Jaana et al. [75]	A measure of clinical information technology sophistication in hospitals	Poor	Not assessed	Not assessed	Adequate (68)	3
	**Sustainability (number of instruments = 3):** the extent to which a newly implemented treatment is maintained or institutionalised within a service setting’s ongoing, stable operations. Commonly used terms: maintenance, continuation, durability, incorporation, integration, institutionalisation, sustained use and routinisation					
Finch et al. [76]	Normalisation Measure Development Questionnaire (NoMAD)	Fair	Fair	Not assessed	Good (23)	7
Elf et al. [77]	Normalisation Measure Development Questionnaire (S-NoMAD)—Swedish version	Fair	Poor	Not assessed	Good (20)	7
Slaghuis et al. [78]	A measurement instrument for sustainability of work practices in long-term care—short version	Poor	Poor	Not assessed	Good (30)	7
	A measurement instrument for sustainability of work practices in long-term care—long version	Poor	Poor	Not assessed	Good (40)	6
Barab et al. [79]	The Levels of Institutionalization (LoIn) scales	Poor	Poor	Not assessed	Good (30)	4

*1. Minimal: > 100 items; 2. adequate: 50–99 items; 3. good: 10–49 items; 4. excellent: < 10 items (Lewis et al. [13])

**One analysis was reported where the 3 scales were included in the same model

***Test-retest reliability procedure not reported therefore unable to score

Underlined rows indicate instruments with multiple scales

## Results

### Selection of studies

**Fig. 1:**
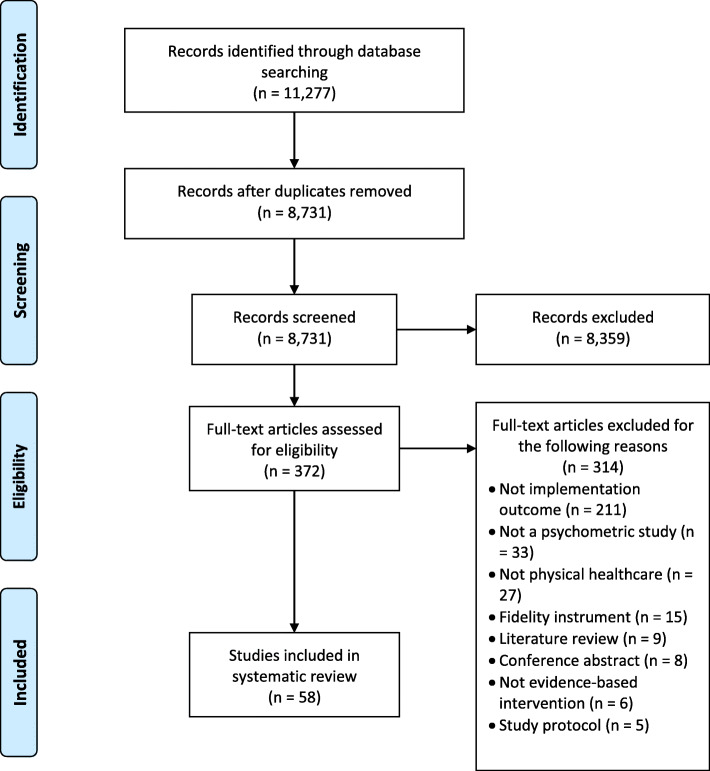
PRISMA flow diagram

A total of 11,277 citations were identified for title and abstract screening, of which 372 citations were considered potentially eligible for inclusion, and full-text publications were retrieved. The total number of excluded publications was 315, where the majority of papers were excluded because they did not assess an implementation outcome (*n* = 212) (see Fig. 1 for PRISMA flowchart).

Of the 372 manuscripts screened, 58 were eligible for inclusion and data were extracted. Fifty-eight manuscripts reported on the measurement properties of 55 implementation outcome instruments, with 65 individual scales. Of the 55 instruments identified, 7 instruments had multiple scales: Evidence-Based Practice Attitude Scale (3 scales: English, Greek and Turkish) [33, 56, 58]; Evidence-Based Practice Questionnaire (3 scales: English, Swedish and Japanese) [27, 28, 55]; Diffusion of Innovation in Long-Term Care (DOI-LTC) measurement battery (2 scales: licensed nurse and certified nursing assistant) [38]; Mind the Gap Scale (2 scales: parent and adolescent) [23]; The Attitudes Related to Trauma-Informed Care (3 scales: 45-, 35- and 10-item) [35]; The Normalisation Measure Development Questionnaire (NoMAD) (2 scales: English and Swedish) [76, 77] and a measurement instrument for sustainability of work practices in long-term care (2 scales: 40- and 30-item) [78].

### Instrument and study characteristics

**Fig. 2:**
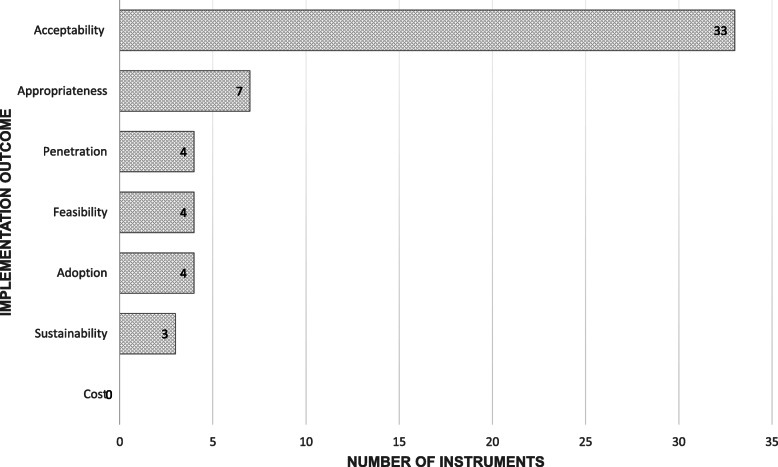
Number of instruments identified across implementation outcomes

The majority of included studies reported measurement properties of instruments that assessed acceptability (*n* = 33) [23–59], followed by appropriateness (*n* = 7) [46, 60–65], adoption (*n* = 4) [66–69], feasibility (*n* = 4) [46, 70, 71, 80], penetration (*n* = 4) [72–75] and sustainability of evidence-based practice (intervention or service) (*n* = 3) [76–79]. No studies of intruments measuring implementation cost were identified (see Fig. 2). The number of studies assessing implementation outcome measures has increased over time, with one study published in 1998 [79], through to eight studies published in 2018/2019 [27, 42, 45, 49, 56, 62, 76, 77]. Studies were predominantly conducted in high-income countries, with the majority conducted in the USA alone (*n* = 22) [25, 29, 30, 35, 37–39, 44, 46, 47, 53, 57, 58, 64, 66, 67]. A small number of studies were conducted in lower-middle-income countries (LMIC), namely Brazil [31, 60] and Iran [52]. See Additional file 2 for total number of implementation outcome instruments across high and low/middle-income countries.

The level of analysis, meaning whether the construct assessed was captured at the level of a consumer, provider or organisation, varied by implementation outcome. Scales assessing acceptability were predominantly geared towards providers. Organisation-level analysis was observed for scales assessing penetration and sustainability, though the overall number of these scales was small. Summary of level of analysis across outcomes as follows:

- Acceptability: consumer = 10 [23, 37, 39, 41, 42, 44, 51, 52, 57, 59], provider = 27 [24–36, 38, 40, 43, 45–50, 53–56, 58]
- Appropriateness: consumer = 3 [61–63], provider = 3 [46, 60, 64], both consumer and provider = 1 [65]
- Adoption: consumer = 1 [66], provider = 3 [67–69]
- Feasibility: provider = 4 [46, 70, 71, 80]
- Penetration: organisation = 4 [72–75]
- Sustainability: provider = 2 [76, 77], organisation = 2 [78, 79]

### Methodological quality

Table 1 presents the global COSMIN scores for reliability, validity and responsiveness, and rank orders the instruments by descending reliability scores. Detailed COSMIN scores for individual measurement properties are presented in Additional file 3. The COSMIN checklist was applied to the study design of complete instruments. Where multiple versions of the same instrument are reported (i.e. different language versions, different number of items, different target audience), we extracted data on these scales individually (see Table 1 and Additional files 2, 3 and 4, where underlined rows indicate multiple eligible scales). Reliability: 62/65 scales reported reliability; 6/62 were rated as ‘excellent’, 2/62 ‘good’, 19/62 ‘fair’ and 35/62 ‘poor’. Validity: 63/65 scales reported validity; 7/63 were rated as ‘excellent’, 4/63 ‘good’, 16/63 ‘fair’ and 36/63 ‘poor’. Responsiveness: 2/65 scales reported responsiveness, both of which were rated as ‘poor’.

### Psychometric quality

Twelve (12/65) scales scored 7 or more (of the maximum possible score = 22), indicating greater psychometric strength on the ConPsy checklist [23, 45, 46, 61, 69, 76–78]. Detailed ConPsy scores for each scale are presented in Additional file 4. Most studies only reported the internal consistency of the scales and only calculated Cronbach’s alpha, hence generally receiving low ConPsy scores. Only 5 scales reported evidence on the stability (or test-retest reliability) of the scale [35, 37, 69]. Forty-six publications did not assess the reliability of the reported instrument, yet they did assess structural or criterion related validity. According to psychometric theory, reliability is a prerequisite for validity and therefore should always be assessed first [81]. Assessment of dimensionality using factor analysis was attempted for 50 scales. Of those, only 2 scales adequately presented both exploratory factor analysis (EFA) and confirmatory factor analysis (CFA) [33, 74]. Both analyses are recommended, where EFA can confirm a theory if the data-at-hand replicate the expected model and CFA can explore the factor structure by testing different models [82]. No studies presented the results with respect to measurement invariance, which in contemporary psychometrics is acknowledged as a vital part of scale evaluation [82].

### Instrument usability

The number of items within each instrument and scale ranged from 4 to 68, with median of 24 (inter-quartile range—IQR 15, 31.5). Six scales contained fewer than 10 items (rated ‘excellent’); 55 scales contained between 10 and 49 items (rated ‘good’) and four scales contained between 50 and 99 items (rated ‘adequate’). See Table 1 for the number of items of each reviewed instrument. There was no significant correlation between usability and COSMIN reliability scores *r*_s_ = .03, *p* = .81; or usability and COSMIN validity scores *r*_s_ = − .08, *p* = .55. We found a small negative correlation between usability and ConPsy scores, which was statistically significant *r*_s_ = − .28, *p* = .03.

## Discussion

### Summary of findings

This systematic review provides a repository of 55 methodologically and psychometrically appraised implementation outcome instruments that can be used to evaluate the implementation of evidence-based practices in physical healthcare settings. We found an uneven distribution of instruments across implementation outcomes, with the majority assessing acceptability. This review represents the first attempt, to our knowledge, to systematically identify and appraise quantitative implementation outcome instruments for methodological and psychometric quality, and usability in physical healthcare settings. Researchers, healthcare practitioners and managers looking to quantitatively assess implementation efforts are encouraged to refer to the instruments identified in this review before developing ad hoc or project-specific instruments.

### Comparison with other studies

Our findings are similar to those reported in Lewis et al.’s systematic review of implementation outcome instruments validated in mental health settings [13]. Whilst our review identified a smaller number of instruments (55 compared with 104), both reviews found uneven distributions of instruments across implementation outcomes, with most instruments identified as measuring acceptability. Lewis et al. suggest the number and quality of instruments relates to the history and degree of theory and published research relevant to a particular construct, noting that ‘there is a longstanding focus on treatment *acceptability* in both the theoretical and empirical literature, thus it is unsurprising that *acceptability* (of the intervention) is the most densely populated implementation outcome with respect to instrumentation’ [13] p.9. Based on our experience of reviewing the instruments included in this review, we also suggest that acceptability is a broader construct that encompasses more variability in definition compared with other constructs, such as penetration and sustainability that are narrower in focus.

Furthermore, the number of studies that assess the measurement properties of implementation outcome instruments increased over time, which mirrors the findings of Lewis et al. [13]. We concur with Lewis et al. [13] that the assessment of implementation cost and penetration may be more suited to formula-based instruments as they do not reflect latent constructs. This is likely to explain why our review found no instruments that assessed implementation costs. It must be noted that whilst we did not identify any implementation cost instruments, previous reviews have [13, 14]. In accordance with instruments in mental health, our review of instruments in physical health identified few studies from lower- and middle-income countries, which highlights the need for further cross-cultural validation studies.

### Methodological and psychometric quality

The COSMIN checklist [20] provides a set of questions to assess the methodological quality of studies of measurement properties of outcome instruments, which is widely used and recognised. The application of the COSMIN checklist was a laborious process that required a skilled understanding of psychometric research. The COSMIN checklist uses the ‘lowest score’ rule to give an overall score for each measurement property. Whilst this is a conservative approach to methodological quality assessment, it penalises authors who assess multiple measurement properties rather than a choice few. For example, an instrument with excellent construct and content validity may be scored poorly if the developers attempt to assess concurrent validity, whilst another measure can score higher for only assessing the first two of these constructs. Further, we applied the newly developed ConPsy checklist to assess the psychometric quality of reviewed instruments. The COSMIN assesses the methodological quality of the studies but does not appraise the psychometric quality/strength of the actual tests and indices which we summarise in the ConPsy. ConPsy scores were generally low. A message that emerges from this appraisal is that better application of modern psychometric theory and method is required of implementation scientists. Coherent use of the instruments identified by this review can help expand their psychometric evidence base. This is a further argument for avoiding the development of new instruments when an existing one offers adequate coverage of the underlying implementation construct.

### Instrument usability

We identified instruments that contained a median of 24 items (IQR 15–32), with a maximum of 68 items. This relatively high number of items raises concerns in terms of potential instrument use by investigators seeking to conduct implementation research or health professionals or managers that wish to evaluate implementation efforts in a busy practice setting. This is a particular concern when multiple implementation outcomes are assessed concurrently. Glasgow et al. advocate the development, validation and use of pragmatic instruments that have a low burden for staff and respondents, i.e. are brief and cheap to use [83]. Whilst the mode of administration can be cheap (e.g. printing an existing validated questionnaire), time is an issue when the questionnaire is long. Item brevity is considered important for implementation outcome measures, as with patient reported outcome measures (PROMs). For example, in clinical settings, Kroenke et al. [84] recommend the use of a brief (PROM) measure, which they “arbitrarily define […] as ‘single digits’ (less than 10 items) and an ultrabrief measure as one to four items”, or that takes less than 1 to 5 min to complete. The tension that arises here is between adequate coverage of the underlying construct of interest (typically achieved through lengthier scales) and usability and good response rates in clinical settings (typically achieved through brevity). The field as a whole could resolve this tension via developing both longer and shorter versions of the same instruments—a tradition in psychometric science for decades. Interestingly, we found a small significant negative correlation between usability and psychometric quality, suggesting that instruments with fewer items identified by this review were more psychometrically sound. We found no evidence of a correlation between usability and methodological quality; however, these results should be interpreted with caution as most instruments fell within the ‘Good’ usability category (i.e. 10–49 items). Furthermore, it must be noted that number of items is a crude measure of usability and efforts are currently underway to develop a concept of how ‘pragmatic’ a measure is that goes beyond the number of items [85, 86].

### Conceptualising implementation outcomes

Proctor et al.’s taxonomy was a useful framework to guide the inclusion of implementation outcome instruments included in this systematic review and enabled comparison of our findings with a published systematic review that focussed on mental health settings [13]. However, we found that the definitions used by authors/instrument developers often differed from those used by Proctor et al., leading to a high number of excluded studies that did not fit the taxonomy. Researchers have highlighted the conceptual overlap among implementation outcomes [13], and this indeed represented a significant challenge in categorising the instruments identified in this review. As highlighted by Proctor et al., the concepts of acceptability and appropriateness overlap. Further efforts are needed to standardise the conceptualisation of implementation outcomes, where this is feasible. A recommendation would be to re-classify ‘intention to use’ as a measure of acceptability or appropriateness rather than adoption.

Another recommendation is to link the development and the use of implementation outcome measures to a theory—such that there is an underlying system of hypotheses about how an intervention and/or implementation strategy is expected to ‘work’, including how different outcomes, i.e. patient, service and implementation, are ‘expected’ to correlate with each other. For instance, to take a long-standing and well-evidenced psychological theory of relevance to understanding human social behaviours, the theory of planned behaviour postulates that intention to engage in a behaviour is determined by the attitudes towards the behaviour, the perceived behavioural control over it and the subjective behavioural norms [87–90]. Following decades of development and use, the theory now offers numerous measures that can be applied to different behaviours and related intentions, and hypotheses regarding how these measures will correlate and interact (including with other variables) to produce a behaviour (or not). This review identified four instruments based on the technology acceptance model [32, 40, 57, 71], which has also informed instruments validated in mental health [91] and community settings [92]. Use of such theory-informed measures allows corroboration of the theory across the settings in which it is applied—which is of direct relevance to the science of implementation. At the same time, it allows practical predictions or explanations to be developed for an observed behaviour—another attribute of direct relevance to the science of implementing an evidence-based intervention. Implementation science has started to develop numerous theoretical strands [4], and it is also beginning to integrate theory in the design of studies and selection of implementation strategies [93, 94]. Coherent use of theory can also inform implementation outcome measurement.

### Strengths and limitations of the study

This is one of the first systematic reviews of implementation outcome instruments to use comprehensive search terms to identify published literature, without language restrictions, and to critically appraise the included studies using the COSMIN checklist. Further, this review uses bespoke psychometric quality criteria that assess the psychometric strength of the instruments (i.e. the ConPsy checklist). There are, however, limitations with this review, which relate to the vast literature that was identified and our capacity for managing it. These were as follows: (1) not using a psychometric search filter, (2) not searching grey literature (as intended *a priori*) and (3) excluding studies where only a sub-scale (as opposed to the entire scale) was eligible for inclusion. Unlike systematic reviews that answer an effectiveness question, the purpose of this systematic review was to identify a repository of instruments; we therefore made the pragmatic decision to limit the review in this way. The use of Proctor et al.’s taxonomy as a guiding framework for the inclusion of implementation outcomes (excluding fidelity instruments) carries the limitation of excluding potentially important instruments measuring outcomes not included in the taxonomy. Use of the framework was necessary to set parameters around already broad inclusion criteria. This review did not evaluate the quality of translation for non-English language versions of the included instruments; this is an important consideration for people who choose to use these instruments and guidelines are available to support researchers with the translation, adaptation and cross-cultural validation of research instruments [95, 96].

### Implications

First and foremost, the identification and selection of implementation outcome instruments should be guided and aligned with the aims of a given implementation project. If more than one implementation outcome is of interest, then multiple instruments, assessing different implementation outcomes, should be applied. That said, if multiple implementation outcomes are of interest, researchers and practitioners should consider the burden to those completing the instruments. It may not be realistic to expect stakeholders to complete multiple instruments (for different implementation outcomes). The number of instruments that researchers/practitioners apply is likely to depend on the pragmatic nature of the instruments. As such, there may be a trade-off between psychometrically robust and pragmatic instruments.

The methodological and psychometric limitations of the studies and instruments identified by this review suggest that implementation outcome measurement needs rapid expansion to address current and future measurement needs, for quantitative implementation studies and hybrid trials. Most instruments to date are context- and intervention-specific, with only the Evidence-Based Practice Attitude Scale found to be validated in both physical [33, 56, 58] and mental health settings [97, 98], although generically applicable measures are starting to emerge [46, 76, 77], and an increased emphasis on pragmatic, short measures are evident in the literature [83]. Further psychometric testing of the instruments identified here, and/or focused instrument development is needed to tailor the instruments to specific intervention and physical health setting requirements. We recommend further research to include cultural adaptation, tailoring and revalidation studies—including outside North America, where the majority of the reviewed evidence has been generated to date, and within LMICs. The ability of the instruments to capture different implementation challenges and to be applied successfully across different healthcare settings will offer further validation evidence.

Further, in light of the state of the evidence, we propose that implementation scientists undertake psychometric validation studies of instruments that capture both similar and also different implementation outcomes. The validation approach of the ‘multitrait-multimethod’ matrix [99] is an avenue that we believe will help deliver measurement advances in the field. In brief, the matrix allows establishment of convergent and discriminant validities via examination of correlations between instruments measuring similar outcomes (e.g. acceptability) vs. instruments measuring different outcomes (e.g. acceptability vs. appropriateness); simultaneously, the matrix also allows examination of different methods of assessing similar outcomes (e.g. survey-based vs. observational assessments). The former application of the matrix will be easier in the shorter-term future given the state of development of the field.

Importantly, we acknowledge the implication that the science is yet to fully address the pragmatic needs of implementation practitioners and frontline providers and managers, who require guidance through the selection of appropriate instruments for their purposes (e.g. for rapid, pragmatic evaluation of the success of an implementation strategy). As in other areas of practice, greater synergy is required and a collaborative approach between implementation scientists and psychometricians and practitioners. Annotated online databases go some way in addressing this need. Collaborative relationships to popularise reasonably robust instruments for use can be implemented through science-practice applied interfaces, such as the role of ‘researcher-in-residence’ [100, 101], which allows implementation scientists to be fully embedded within healthcare organisations. Also, wider academic-clinical partnerships, such as the model of the Academic Health Science Centres [102], which has gained traction in many countries in recent years, allows smoother interfaces between scientific departments (usually hosted by universities) and clinical departments (usually hosted by hospital or similar healthcare provision organisations).

Efforts have been made to promote the use of implementation outcome instruments via repositories [16]. One online database relies on crowdsourcing, where instrument developers proactively add their publication to the repository [103]. Another database provides implementation outcome instruments specific to mental health settings, to fee-paying members of the Society for Implementation Research Collaboration [103]. These databases have quality and access limitations. To address these, a repository including all the instruments identified in this review will be made freely available online from the Centre for Implementation Science at King’s College London [104]. The repository is due to be launched in October 2020.

## Conclusions

This review provides the first repository of 55 implementation outcome instruments, appraised for methodological and psychometric quality, and their usability relevant to physical health settings. Psychometrically robust instruments can be applied in implementation programme evaluation and health research to promote consistent and comparable implementation evaluations. Rather than developing ad hoc instruments, we encourage further psychometric research on existing instruments with promising evidence.

## Supplementary information

**Additional file 1.** ConPsy checklist scoring guidance.

**Additional file 2.** Characteristics of included studies.

**Additional file 3.** COSMIN scores.

**Additional file 4.** ConPsy scores.

## Data Availability

The data extracted for this systematic review is provided in the Additional files.

## Abbreviations

COSMIN: COnsensus-based Standards for the selection of health Measurement INstruments checklist
ConPsy: Contemporary Psychometrics checklist
SIRC: Society for Implementation Research Collaboration
PROSPERO: Prospective Register of Systematic Reviews
PRISMA: Preferred Reporting Items for Systematic Reviews and Meta-Analyses statement
OpenGrey: System for Information on Grey Literature in Europe
NoMAD: Normalisation Measure Development Questionnaire
LMIC: Lower-middle income countries
EFA: Exploratory factor analysis
CFA: Confirmatory factor analysis
IQR: Inter-quartile range
PROMs: Patient reported outcome measures
