# Supplementary material for: Implementation outcome instruments for use in physical healthcare settings: a systematic review
Source: Implement Sci. 2020 Aug 18;15:66. doi: 10.1186/s13012-020-01027-6 (PMC7433178; doi:10.1186/s13012-020-01027-6)
Supplement: Supplementary file 1 — Additional file 1. ConPsy checklist scoring guidance. [file 13012_2020_1027_MOESM1_ESM.docx]

**Additional file 1. ConPsy checklist scoring guidance**

| **RELIABILITY up to 5 points** |
| --- |
| **Internal consistency**: up to **3** points |
| - **IIC/ITC**: 1 point for either IIC or ITC within the appropriate range (0.25 to 0.80) |
| - **a (or other equivalent coefficient)**: 1 point for dimension alpha >0.65 (smallest counts) |
| 2 if dimension alpha>0.85 (smallest counts) |
| *IIC: inter item correlation; TIC: Item total correlation; a: Cronbach’s alpha* |
| **Test retest** OR **Inter-rater**: up to **2** points (interrater is to be added later on, to compare only with scales that actually needed interrater to avoid artificially augmented scores) |
| - **Item** level: 1 point if correct kappa (or equivalent method) at least 0.4 for all items |
| or if % agreement at least 70% for all items |
| or ICCs per item at least 0.7 (continuous items) |
| - **Factor** (subscale) level: 1 point if ICCs at least 0.7 for all subscale scores (or correlation >0.7 along with a means of medians test-***half*** *points* *if correlation only*) |
| *ICC: Intraclass Correlation Coefficient* |
| **VALIDITY up to 5 points** |
| - **Content**: 1 point for content (experts’) validity - **Face:** 1 point for face validity (target population opinion) - **Convergent**: 1 point for convergent validity reported with correlations of 0.5 or above (larger reported will do) - **Discriminant:**  1 point for discriminant validity (up to 0.5 correlations) - **Discriminative**: 1 point for discriminative validity (that is difference in the scores between groups that complies with the literature) |
|  |
| **FACTOR ANALYSIS** **up to 12** |
| - **Adequacy** **measures**: 1 point if KMO or Bartlett is satisfactory in EFA - **Kaiser** **criterion**: 1 point for reporting eigenvalues or scree plot in EFA - **VE**/**PA**: 1 variance explained more than 50% or parallel analysis indicating the number of factors in EFA - **Loadings**: 1 point if all main loadings are >0.4 in EFA - **GOF**-EFA: 1 point for each index that reaches its desired value in EFA (eg RMSEA, TLI, CFI, x^2^)-up to 4 points - **GOF**-CFA :1 point for each index that reaches its desired value in CFA (RMSEA, TLI, CFI, x^2^)-up to 4 points |
| **Minus 1 point if common factor model (ie spss or amos) was used for less than 5 points ordinal scales, rather than item factor analysis** |
| **Minus 1 point if 2-item factors or more than 20% cross loadings were present.** |
| *KMO: Kaiser-Meyer-Olkin criterion; EFA: Exploratory Factor analysis; CFA: Confirmatory Factor analysis; VE: variance explained; PA: parallel analysis*, *GOF: goodness of fit,* *RMSEA: Root mean square error approximation, TLI: Tucker Lewis Index, CFI: Comparative Fit Index* |
