# Supplementary material for: Implementation outcome instruments for use in physical healthcare settings: a systematic review
Source: Implement Sci. 2020 Aug 18;15:66. doi: 10.1186/s13012-020-01027-6 (PMC7433178; doi:10.1186/s13012-020-01027-6)
Supplement: Supplementary file 2 — Additional file 2. Characteristics of included studies. [file 13012_2020_1027_MOESM2_ESM.docx]

**Additional file 2. Characteristics of included studies**

| **Reference** | **Implementation Outcome**  Name of measurement instrument or instrument description | **Country** | **Sample** | | | | **Evidence-based intervention (EBI) or Service** |
| --- | --- | --- | --- | --- | --- | --- | --- |
|  |  |  | **Size** | **Gender** | **Age** | **Ethnicity** |  |
|  | **Acceptability (number of instruments=33)** | | | | | | |
| Shaw et al. (2007) | The Mind the Gap Scale-Adolescent version | UK | Total: 587 Adolescents: 301 Parents: 286 | Adolescents: ratio of Male to Female 1:1.5 Parents: Female: 83.2% | Adolescents:  Median: 14.2; Range: 10.9-18  Parents: Median: 41.5; Range: 27-67 | Caucasian (adolescents): 91% White/European: 95% | Service |
|  | The Mind the Gap Scale-Parent version |  |  |  |  |  |  |
| Dow et al. (2013) | The Person-Centred Health Care for Older Adults (PCHCOA) Survey | Australia | 1428 | Female: 1228 (86%) Male: 171 (12%)  Not stated: 29 (2%) | Not reported | Not reported | Service |
| Dykes et al. (2007) | The Impact of Health Information Technology (I-HIT) Scale | USA | 1760 | Female: 91.3% (of the non-missing 1079) | Not reported | Not reported | Service |
| Brehaut et al. (2010) | Ottawa acceptability of decision rules instrument (OADRI) | Australasia, Canada, UK and USA | 1297 | Not reported | Not reported | Not reported | Service |
| Tomotaki et al. (2018) | Evidence-Based Practice Questionnaire (EBPQ‐J)-Japanese version | Japan | Total: 533  108 (test-retest) | Not reported | 20-29: 262 (49.2%) 30-39: 142 (26.6%) 40-49: 81 (15.2%) 50-59: 33 (6.2%) 60: 9 (1.7%) Unknown: 6 (1.1%) | Not reported | EBI |
| Upton et al. (2006) | Evidence-Based Practice Questionnaire (EBPQ) | UK | 751 | Female: 90.2%  Male: 9.8% | 30-39 years: 43% | Not reported | EBI |
| Bhor et al. (2006) | A Scale to assess attitudes of health care administrators toward the use of e-mail communication between patients and physicians | USA | 128 | Female: 108 (86.40%) Male: 17 (13.60%) | 18-24: 1 (0.86%)  25-29: 3 (2.56%)  30-34: 10 (8.55)  35-44: 29 (24.79)  45-54: 54 (46.15%)  55-64:20 (17.09%) | Native American: 1 (0.83%)  Asian/Pacific Islander: 2 (1.65%)  Black: 2 (1.65%)  Hispanic: 5 (4.13%)  White: 111 (91.74%) | EBI |
| Phansalkar et al. (2008) | Instrument for assessing clinicians’ perceptions about use of computerized protocols | USA | Total: 240  Nurses: 132 (55%) Physicians: 53 (22.1%) Respiratory therapists: 55 (22.9%) | Not reported | Not reported | Not reported | EBI |
| Oliveira et al. (2011) | CARDIOSATIS-Team scale | Brazil | 152 | Not reported | Mean: 40 (median: 38.5) | Not reported | Service |
| Wu et al. (2008) | Healthcare professionals' intention to use an adverse event reporting system | Taiwan | 290 | Female: 235 (81%)  Male: 55 (19%) | Not reported | Not reported | Service |
| Melas et al. (2012) | The Evidence-Based Practice Attitude Scale (EBPAS)-Greek version | Greece | 604  Medical doctors: 534  Nurses: 70 | Female: 257 (35%) Male: 347 (65%) | Mean: 36.51 ± 7.9; Range: 26-64 | Not reported | EBI |
| Brouwers et al. (2004) | Practitioner Feedback Questionnaire | Canada | 488 | Not reported | Not reported | Not reported | Service |
| Baker et al. (2016) | The Attitudes Related to Trauma-Informed Care (ARTIC-45) | USA | Total (service providers): 760 595 human services, community-based mental health or health care;  165 worked in schools | Female: 83% | > 18 | White and non-Latino: 691 (91.2%) Black or African American: 36 (4.7%) Asian: 12 (1.6%) American Indian or Alaska native: 9 (1.2%) Biracial or multiracial: 10 (1.3%) Hispanic or Latino of any race: 39 (5.1%) | Service |
|  | The Attitudes Related to Trauma-Informed Care (ARTIC-35) Scale |  |  |  |  |  |  |
|  | The Attitudes Related to Trauma-Informed Care (ARTIC-10) Scale-Short version |  |  |  |  |  |  |
| Vanneste et al. (2013) | A survey measuring acceptance of BelRAI, a web-based system enabling person-centred recording and data sharing across care settings. | Belgium | 282 | Female: 216 (76.60%) Male: 66 (23.4%) | 20-29: 69 (24.47%) 30-39: 85 (30.14%) 40-49: 68 (24.11%) >50: 60 (21.28%) | Not reported | Service |
| Bakas et al. (2009) | A rating form measuring the satisfaction of the Telephone Assessment and Skill-Building Kit (TASK) intervention. | USA | 40 | Female: 73% | Not reported | Caucasian: 73% African American: 25% | EBI |
| McConnell et al. (2012) | Diffusion of Innovation in Long-Term Care (DOI-LTC) measurement battery-version for certified nursing assistants | USA | Total: 199  Registered nurse: 50  Licensed practical nurse: 45  Certified nursing assistant: 104 | Registered nurse: Female: 94.9%; Male: 5.1%  Licensed practical nurse: Female: 94.5%; Male: 5.5%Certified nursing assistant: Female: 93.6%; Male: 6.4% | Registered nurse: >50: 36.4%  Licensed practical nurse: >50: 34.4%  Certified nursing assistant: >50: 18.1% | Registered nurses: African American: 6%  Asian: 0% Caucasian: 92% Hispanic: 0%  Other: 0% Missing: 2%  Licensed practical nurses: African American: 33.3% Asian: 0% Caucasian: 57.8% Hispanic: 0%  Other: 2.2% Missing: 6.7%  Certified nursing assistants: African American: 39.2% Asian: 5.9% Caucasian: 52.9% Hispanic: 0%  Other: 3.9% Missing: 0% | EBI |
|  | Diffusion of Innovation in Long-Term Care (DOI-LTC) measurement battery-version for licensed nurses |  |  |  |  |  |  |
| Atkinson (2007) | A Questionnaire to Measure Perceived Attributes of eHealth Innovations | USA | 193 | Female: 88 Male: 98 | 18-21: 130 22-25: 32 26-29: 6 30-39: 10 40+: 7 | African American: 7 White: 141 Hispanic/Latino: 14 Asian/Pacific Islander: 6 Other: 2 | Service |
| Gagnon et al. (2012) | A questionnaire based on the Technology Acceptance Model (TAM) | Spain | 93 | Women: 76 (81.72%) Men: 17 (18.28%) | <30: 24 (25.81%) 30–39: 21 (22.58%) 40–49: 30 (32.26%) 50–59: 15 (16.13%) >60: 3 (3.23%) | Not reported | EBI |
| Ferrando et al. (2010) | A questionnaire to measure convenience and satisfaction with a new internet-based tool for oral anticoagulation therapy telecontrol | Spain | 98 | Female: 43 Male: 55 | Mean: 62.5 (SD: 16.4)  Median: 61; Range: 18-90 | Not reported | EBI |
| Wilkinson et al. (2018) | A survey measuring attitudes towards biomedical HIV prevention | Australia | 462 | Male: 455 (98.5%) Trans man: 2 (0.4%) Non-binary/gender queer: 5 (1.1%) | Not reported | Not reported | EBI |
| Adu et al. (1999) | A questionnaire measuring pharmacists and physician’s attitudes to antibiotic policies | Australia | Total: 943 Physicians: 702  Pharmacists:241 | Not reported | Not reported | Not reported | Service |
| Abetz et al. (2005) | Cancer Therapy Satisfaction Questionnaire (CTSQ) | USA | Total: 95  Patients: 70 Nurses and oncologists: 25 | Patients: Female: 53 (76%) Nurses and oncologists: 5 (20%) | Patients: 61.6 ± 10.5; Range: 38-76 Nurses and Oncologists: Not reported | Countries of origin or nationalities: Patients: Not reported  USA: 60  UK: 5 France: 5  Nurses and oncologists:  USA: 19 UK: 3 from France: 3 | EBI |
| Blumenthal et al. (2018) | Physiotherapy Mobile Acceptance Questionnaire (PTMAQ) | Canada | 76 | Female: 356 (98.6%) Male: 5 (1.4%) | <25: 27 (35.5%) 25-34: 26 (34.2%) 35-44: 13 (17.1%) 45-54: 5 (6.6) >55: 5 (6.6) | Not reported | Service |
| Weiner et al (2017)** | Acceptability of Intervention Measure (AIM) | USA | Study 1: 63 Study 2: 326  Study 3: 192 | Study 1: Female: 32% Male: 68%  Study 2: Female: 71% Male: 28% Non-binary: 1%  Study 3: Female: 73% Male: 26% Non-binary: 1% | Not reported | Study 1: Caucasian: 94% African American: 3% Asian: 3% Hispanic: 11%  Study 2: Caucasian: 91% African American: 5% Asian: 2% Hispanic/Latino/Spanish origin: 4% Other: 2%  Study 3: Caucasian: 94% African American: 2% Asian: 2% Hispanic/Latino/Spanish origin: 5% Other: 2% | EBI |
| Unni et al. (2016) | A survey measuring satisfaction with Electronic health records | USA | 90 | Not reported | Not reported | Not reported | Service |
| Aggelidis et al. (2012) | End user computing satisfaction (EUCS) survey | Greece | 283 | Female: 186 (65.7%) Male: 97 (34.3%) | 20–30: 18 (6.4%) 31–35: 40 (14.1%) 36–40: 70 (24.7%) 41–50: 129 (45.6%) >50: 26 (9.2%) | Not reported | Service |
| El-Den et al. (2018) | Perinatal Depression (PND) Attitudes and Screening Acceptability Questionnaire (PASAQ) | Australia | 153 | Female: 127 (83.6%) Male: 26 (16.4%) | Range: 24-67 | Not reported | EBI |
| Kramer et al. (2014) | A generic questionnaire to detect physicians’ willingness to implement complex medical interventions | Germany | 181 | Female: 74 (41%) Male: 107 (59%) | Mean: 51.9 (SD:7.6) <40: 11 (6%) 41-50: 64 (35%) >51: 106 (59%) | Not reported | EBI |
| Frandes et al. (2017) | An instrument assessing mobile technology acceptability in diabetes self-management | Romania | 103 | Female: 52 (50.5%) | Median: 37; Range: 18-65 | Not reported | EBI |
| Rasoulzadeh et al. (2017) | A questionnaire measuring acceptance of creating a nurses' health monitoring system | Iran | 586 | Female: 441 (75.3%) Male: 145 (24.7%) | <25: 93 (15.9%) 25-35: 260 (44.4%) 35-45: 153 (26.1%) 45-55: 77 (13.1%) >55: 3 (0.5%) | Not reported | Service |
| Sockolow et al. (2011) | Electronic Health Record Nurse Satisfaction (EHRNS) survey | USA | Total: 52 (test); 45 (retest)  Sample of clinicians at a site with an EHR: 37 (test), 32 (retest) Sample of clinicians at a paper-based site: 15 (test 1), 13 (retest) | Not reported | Pilot test:  Mean: 26  Both sites:  Mean: 49 | Not reported | EBI |
| Johnston et al. (2002) | A questionnaire assessing physicians' attitudes towards the computerization of clinical practice | Hong Kong | 373 | Physicians in individual practices:  Female: 56 (14.75%) Male: 317 (85.25%) Physicians in corporate practice: No socio-demographic information | Physicians in individual practices:  21-30: 7 (1.9%) 31-40: 86 (23.1%) 41-50: 117 (31.4%) 51-60: 97 (26%) 61-70: 56 (15%) >70: 10 (2.7%)  Physicians in corporate practice: no socio-demographic information reported | Not reported | Service |
| Bernhardsson et al. (2013) | Evidence-Based Practice (EBP) questionnaire-Swedish version | Sweden | Total: 52    Validity test: 10  Reliability test: 42 | Validity test: Women: 8 Men:2  Reliability test: Women:5 Men: 37 | Validity test: Median:35  Reliability test:  20-29: 3  30-39: 15  40-49: 10  50-59: 10  60+: 4 | Not reported | Service |
| Yildiz et al. (2018) | Evidence-Based Practice Attitude Scale (EBPAS-50)-Turkish version | Turkey | 250 | Female: 241 (96.4%) Male: 9 (3.6%) | Mean: 33.80 (SD=6.83) | Not reported | EBI |
| Bevier et al. (2014) | Questionnaire of three scoring items for current treatment satisfaction and factors of both clinical trial participation motivations and technology acceptance model | USA | 36 | Female: 25 Male: 11 | Mean: 46.6 (SD:12.5) | Not reported | EBI |
| Silver Wolf et al. (2014) | Evidence-Based Practice Attitude Scale (EBPAS) | USA | 1260 | Female: 59% Male: 41% | Mean: 35 (SD=11); Range: 19-73 | White: 74% African American: 17% Other: <5% | EBI |
| Steed et al. (2008) | Acceptability of Continuous Glucose Monitoring Devices (ACGMD) questionnaire | UK | 19 | Study 2: Female: 53%, Male: 47% | Study 1:  Range: 25-56  Study 2:  Mean: 41 | Not reported | EBI |
|  | **Appropriateness (number of instruments=7)** | | | | | | |
| Diego et al. (2016) | A questionnaire to measure the attitude of anesthesiologists and residents regarding the use of the checklist in the perioperative period | Brazil | 459 | Female: 45%  Male: 55% | Mean: 40.7 | Not reported | EBI |
| Park et al. (2016) | A questionnaire measuring motivational factors for using wearable healthcare devices | South Korea | 877 | Female: 379 (43.2%) Male: 498 (56.8%) | 20-29: 271 (30.9%) 30-39: 351 (40%) 40-49: 171 (19.5%) 50-59: 51 (5.8%) >59: 33 (3.8%) | Not reported | Service |
| Razmak et al. (2018) | A Techno-humanist model for e-health adoption of innovative technology | Canada | 325 | Female: 60.6% Male: 39.4% | <35: 59.7% 35-54: 26.8% >55: 13.5% | Not reported | Service |
| Joice et al. (2012) | Perceived usefulness of a stroke workbook-based intervention measure | UK | 59 | Female: 26 Male: 33 | Mean: 68.22 (SD: 11.16) ; Range: 37-85 | Not reported | EBI |
| Xiao et al. (2014) | Baylor EHR UX survey | USA | 1301 | Not reported | Not reported | Not reported | Service |
| Weiner et al (2017) | Intervention Appropriateness Measure (IAM) | USA | Study 1: 63 Study 2: 326  Study 3: 192 | Study 1: Female: 32% Male: 68%  Study 2: Female: 71% Male: 28% Non-binary: 1%  Study 3: Female: 73% Male: 26% Non-binary: 1% | Not reported | Study 1: Caucasian: 94% African American: 3% Asian: 3% Hispanic: 11% Study 2: Caucasian: 91% African American: 5% Asian: 2% Hispanic/Latino/Spanish origin: 4% Other: 2% Study 3: Caucasian: 94% African American: 2% Asian: 2% Hispanic/Latino/Spanish origin: 5% Other: 2% | EBI |
| King et al. (2017) | The Portal Survey on Satisfaction and Impact on Care | Canada | Total: 23  18 caregivers  5 service providers (for interviews and focus groups only) | Caregivers: Female: 15 (83%) Male: 3 (17%) | Caregivers: 20-34: 4 (22%) 35-49: 8 (44%) 50-64: 6 (33%) | Not reported | Service |
|  | **Adoption** **(number of instruments=4)** | | | | | | |
| Nydegger et al. (2017) | Strength of Implementation Intentions Scale (SIIS) for condom use | USA | Baseline: 590 One year follow-up: 284 | Baseline: Male: 425 (72.03%) Female: 158 (26.78%) Missing: 7 (1.19%) | Baseline: Mean:30.68 (SD=10.45); Range 18-62 | Baseline: White (non-Hispanic): 176 (29.83%) Hispanic (not mixed): 298 (50.51%) Other racial/ethnic minority or mixed: 62 (10.51%) Missing: 54 (9.15%) | EBI |
| Everson et al. (2014) | American Hospital Association IT (AHA-IT) Supplement Survey | USA | 2331 | Not reported | Not reported | Not reported | EBI |
| Malo et al. (2012) | A questionnaire evaluating nurses’ intention to use an electronic medical charting system | Canada | 62 | Female: 45 (74%) Male: 16 (26%) | <31: 16 (26%) 31-40: 19 (31%) >40: 21 (34%) | Not reported | Service |
| Kaltenbrunner et al (2017) | Lean in Healthcare Questionnaire (LiHcQ) | Sweden | Total: 386  243 for CFA | Female: 333 Male: 49 | Mean: 50 (SD=10) Median: 51; Range: 43-58 | Not reported | Improvement methodology |
|  | **Feasibility (number of instruments=4)** | | | | | | |
| Garcia-Smith et al (2013) | Instrument to test the Clinical Information Systems Success Model (CISSM) | USA | 234 | Female: 91% | Mean: 38 (SD: 10.3) | White non-Hispanic: 62% | EBI |
| Schnall et al. (2011) | Technology Acceptance Survey | USA | 94 | Female: 80.9% Male: 19.1% | Range: 20-40 | Black: 41.5% Hispanic: 38% White: 34.1% | Service |
| Windsor et al. (2013) | The Smoking Cessation and Reduction in Pregnancy Treatment (SCRIPT) Adoption Scale | USA | 85 | Not reported | Not reported | Not reported | EBI |
| Weiner et al (2017) | Feasibility of Intervention Measure (FIM) | USA | Study 1: 63 Study 2: 326  Study 3: 192 | Study 1: Female: 32% Male: 68%  Study 2: Female: 71% Male: 28% Non-binary: 1%  Study 3: Female: 73% Male: 26% Non-binary: 1% | Not reported | Study 1: Caucasian: 94% African American: 3% Asian: 3% Hispanic: 11%  Study 2: Caucasian: 91% African American: 5% Asian: 2% Hispanic/Latino/Spanish origin: 4% Other: 2%  Study 3: Caucasian: 94% African American: 2% Asian: 2% Hispanic/Latino/Spanish origin: 5% Other: 2% | EBI |
|  | **Penetration (number of instruments=4)** | | | | | | |
| Grooten et al. (2019) | The Scaling Integrated Care in Context (SCIROCCO) tool | Spain, Sweden, Italy, Czech Republic, UK, Denmark, France, Germany, the Netherlands. | 67  28 for convergent validity | Not reported | Not reported | Not reported | EBI |
| Slaghuis et al. (2013) | A measurement instrument for spread of quality improvement in healthcare | The Netherlands | Total: 112  Former project leaders: 45 (42%) Team members: 67 (58%) | Female: 80 (77%) Male: 32 (23%) | Not reported | Not reported | EBI |
| Flanagan et al. (2007) | The Prevention and Control of Antimicrobial resistance (PACAR) scale | USA | 448 | Not reported | Not reported | Not reported | EBI |
| Jaana et al. (2005) | A measure of clinical information technology sophistication in hospitals | USA and Canada | 74 hospitals (USA) | Not reported | Not reported | Not reported | Service |
|  | **Sustainability (number of instruments=3)** | | | | | | |
| Finch et al. (2018) | Normalisation Measure Development Questionnaire (NoMAD) | UK | 831 | Not reported | Not reported | Not reported | EBI |
| Elf et al. (2018) | Normalisation Measure Development Questionnaire (S-NoMAD) - Swedish version | Sweden | 144 | Not reported | Not reported | Not reported | EBI |
| Slaghuis et al. (2011) | A measurement instrument for sustainability of work practices in long-term care-Short version | USA | 112 | Female: 80 (77%) Male: 24 (23%) | Mean: 45.2 (SD: 9.3); Range: 19-62 | Not reported | EBI |
|  | A measurement instrument for sustainability of work practices in long-term care-Long version |  |  |  |  |  |  |
| Barab et al. (1998) | The Levels of Institutionalization (LoIn) scales | USA | 132 | Not reported | Not reported | Not reported | Service |
| NOTE: Underlined rows indicate instruments with multiple versions. | | | | | | | |
