# Supplementary material for: Implementation outcome instruments for use in physical healthcare settings: a systematic review
Source: Implement Sci. 2020 Aug 18;15:66. doi: 10.1186/s13012-020-01027-6 (PMC7433178; doi:10.1186/s13012-020-01027-6)
Supplement: Supplementary file 3 — Additional file 3. COSMIN scores. [file 13012_2020_1027_MOESM3_ESM.docx]

| **Additional file 3. COSMIN scores** | | | | | | | | | | |
| --- | --- | --- | --- | --- | --- | --- | --- | --- | --- | --- |
| **Reference** | **Implementation Outcome**  Name of measurement instrument or instrument description | **Reliability** | | | **Validity** | | | | | **Responsiveness** |
|  |  | **Internal Consistency** | **Reliability** | **Measurement Error** | **Content Validity** | **Structural Validity** | **Hypotheses Testing** | **Cross-cultural Validity** | **Criterion Validity** |  |
|  | **Acceptability (number of instruments=33)** | | | | | | | | | |
| Shaw et al. (2007) | The Mind the Gap Scale-Adolescent version | excellent | Not assessed | Not assessed | excellent | excellent | Not assessed | Not assessed | Not assessed | Not assessed |
|  | The Mind the Gap Scale-Parent version | excellent | Not assessed | Not assessed | excellent | excellent | Not assessed | Not assessed | Not assessed | Not assessed |
| Dow et al. (2013) | The Person-Centred Health Care for Older Adults (PCHCOA) Survey | excellent | Not assessed | Not assessed | excellent | excellent | Not assessed | Not assessed | Not assessed | Not assessed |
| Dykes et al. (2007) | The Impact of Health Information Technology (I-HIT) Scale | excellent | Not assessed | Not assessed | good | excellent | good | Not assessed | excellent | Not assessed |
| Brehaut et al. (2010) | Ottawa acceptability of decision rules instrument (OADRI) | excellent | Not assessed | Not assessed | excellent | excellent | poor | Not assessed | Not assessed | Not assessed |
| Tomotaki et al. (2018) | Evidence-Based Practice Questionnaire (EBPQ‐J) - Japanese version | good | good | Not assessed | fair | fair | Not assessed | fair | good | Not assessed |
| Upton et al. (2006) | Evidence-Based Practice Questionnaire (EBPQ) | fair | Not assessed | Not assessed | excellent | fair | Not assessed | Not assessed | Not assessed | Not assessed |
| Bhor et Mason (2006) | A Scale to assess attitudes of health care administrators toward the use of e-mail communication between patients and physicians | fair | Not assessed | Not assessed | excellent | fair | Not assessed | Not assessed | Not assessed | Not assessed |
| Phansalkar et al. (2008) | Instrument for assessing clinicians’ perceptions about use of computerized protocols | fair | Not assessed | Not assessed | excellent | fair | Not assessed | Not assessed | Not assessed | Not assessed |
| Oliveira et al. (2011) | CARDIOSATIS-Team scale | fair | Not assessed | Not assessed | poor | fair | Not assessed | Not assessed | Not assessed | Not assessed |
| Wu et al. (2008) | Healthcare professionals' intention to use an adverse event reporting system | fair | Not assessed | Not assessed | poor | fair | fair | Not assessed | Not assessed | Not assessed |
| Melas et al. (2012) | The Evidence-Based Practice Attitude Scale (EBPAS)-Greek version | fair | Not assessed | Not assessed | Not assessed | fair | fair | poor | Not assessed | Not assessed |
| Brouwers et al. (2004) | Practitioner Feedback Questionnaire | fair | Not assessed | Not assessed | excellent | poor | Not assessed | Not assessed | Not assessed | Not assessed |
| Baker et al. (2016) | The Attitudes Related to Trauma-Informed Care (ARTIC-45) | fair | fair | Not assessed | excellent | fair | fair | Not assessed | poor | Not assessed |
|  | The Attitudes Related to Trauma-Informed Care (ARTIC-35) Scale | fair | fair | Not assessed | excellent | fair | fair | Not assessed | poor | Not assessed |
|  | The Attitudes Related to Trauma-Informed Care (ARTIC-10) Scale-Short version | poor | fair | Not assessed | excellent | poor | fair | Not assessed | poor | Not assessed |
| Vanneste et al. (2013) | A survey measuring acceptance of BelRAI, a web-based system enabling person-centred recording and data sharing across care settings. | fair | Not assessed | Not assessed | Not assessed | fair | fair | poor | Not assessed | Not assessed |
| Bakas et al. (2009) | A rating form measuring the satisfaction of the Telephone Assessment and Skill-Building Kit (TASK) intervention. | poor | poor | poor | excellent | Not assessed | Not assessed | Not assessed | Not assessed | Not assessed |
| McConnell et al. (2012) | Diffusion of Innovation in Long-Term Care (DOI-LTC) measurement battery-version for certified nursing assistants | poor | Not assessed | Not assessed | excellent | Not assessed | Not assessed | Not assessed | Not assessed | Not assessed |
|  | Diffusion of Innovation in Long-Term Care (DOI-LTC) measurement battery-version for licensed nurses | poor | Not assessed | Not assessed | excellent | Not assessed | Not assessed | Not assessed | Not assessed | Not assessed |
| Atkinson (2007) | A Questionnaire to Measure Perceived Attributes of eHealth Innovations | good | poor | Not assessed | Not assessed | good | Not assessed | Not assessed | Not assessed | Not assessed |
| Gagnon et al. (2012) | A questionnaire based on the Technology Acceptance Model (TAM) | poor | Not assessed | Not assessed | excellent | Not assessed | fair | Not assessed | Not assessed | Not assessed |
| Ferrando et al. (2010) | A questionnaire to measure convenience and satisfaction with a new internet-based tool for oral anticoagulation therapy telecontrol | good | poor | Not assessed | good | good | fair | Not assessed | good | Not assessed |
| Wilkinson et al. (2018) | A survey measuring attitudes towards biomedical HIV prevention | poor | Not assessed | Not assessed | Not assessed | fair | Not assessed | Not assessed | Not assessed | Not assessed |
| Adu et al. (1999) | A questionnaire measuring pharmacists and physician’s attitudes to antibiotic policies | fair | poor | poor | excellent | fair | poor | Not assessed | Not assessed | poor |
| Abetz et al. (2005) | Cancer Therapy Satisfaction Questionnaire (CTSQ) | poor | poor | poor | fair | fair | poor | poor | poor | poor |
| Blumenthal et al. (2018) | Physiotherapy Mobile Acceptance Questionnaire (PTMAQ) | poor | Not assessed | Not assessed | excellent | poor | poor | Not assessed | Not assessed | Not assessed |
| Weiner et al. (2017)* | Acceptability of Intervention Measure (AIM) | poor | poor | Not assessed | good | poor | Not assessed | Not assessed | Not assessed | Not assessed |
| Unni et al. (2016) | A survey measuring satisfaction with Electronic health records | poor | Not assessed | Not assessed | good | poor | Not assessed | Not assessed | Not assessed | Not assessed |
| Aggelidis et al. (2012) | End user computing satisfaction (EUCS) survey | poor | Not assessed | Not assessed | excellent | poor | fair | Not assessed | Not assessed | Not assessed |
| El-Den et al. (2018) | Perinatal Depression (PND) Attitudes and Screening Acceptability Questionnaire (PASAQ) | poor | Not assessed | Not assessed | Not assessed | poor | Not assessed | Not assessed | Not assessed | Not assessed |
| Kramer et al. (2014) | A generic questionnaire to detect physicians’ willingness to implement complex medical interventions | poor | poor | Not assessed | poor | poor | fair | Not assessed | Not assessed | Not assessed |
| Frandes et al. (2017) | An instrument assessing mobile technology acceptability in diabetes self-management | poor | Not assessed | Not assessed | excellent | poor | Not assessed | Not assessed | Not assessed | Not assessed |
| Rasoulzadeh et al. (2017) | A questionnaire measuring acceptance of creating a nurses' health monitoring system | poor | Not assessed | Not assessed | poor | Not assessed | Not assessed | Not assessed | Not assessed | Not assessed |
| Sockolow et al. (2011) | Electronic Health Record Nurse Satisfaction (EHRNS) survey | poor | poor | Not assessed | poor | Not assessed | Not assessed | Not assessed | Not assessed | Not assessed |
| Johnston et al. (2002) | A questionnaire assessing physicians' attitudes towards the computerization of clinical practice | poor | Not assessed | Not assessed | poor | fair | Not assessed | Not assessed | Not assessed | Not assessed |
| Bernhardsson et al. (2013) | Evidence-Based Practice (EBP) questionnaire | poor | poor | Not assessed | poor | Not assessed | Not assessed | poor | Not assessed | Not assessed |
| Yildiz et al. (2018) | Evidence-Based Practice Attitude Scale (EBPAS-50) - Turkish version | poor | poor | Not assessed | Not assessed | fair | Not assessed | poor | excellent | Not assessed |
| Bevier et al. (2014) | Questionnaire of three scoring items for current treatment satisfaction and factors of both clinical trial participation motivations and technology acceptance model | poor | Not assessed | Not assessed | Not assessed | Not assessed | Not assessed | Not assessed | Not assessed | Not assessed |
| Silver Wolf et al. (2014) | Evidence-Based Practice Attitude Scale (EBPAS) | Not assessed | Not assessed | Not assessed | Not assessed | excellent | Not assessed | Not assessed | Not assessed | Not assessed |
| Steed et al. (2008) | Acceptability of Continuous Glucose Monitoring Devices (ACGMD) questionnaire | Not assessed | Not assessed | Not assessed | fair | Not assessed | Not assessed | Not assessed | Not assessed | Not assessed |
|  | **Appropriateness (number of instruments=7)** | | | | | | | | | |
| Diego et al. (2016) | A questionnaire to measure the attitude of anesthesiologists and residents regarding the use of the checklist in the perioperative period | fair | Not assessed | Not assessed | Not assessed | good | good | Not assessed | good | Not assessed |
| Park et al. (2016) | A questionnaire measuring motivational factors for using wearable healthcare devices | fair | Not assessed | Not assessed | excellent | fair | fair | Not assessed | Not assessed | Not assessed |
| Razmak et al. (2018) | A Techno-humanist model for e-health adoption of innovative technology | fair | Not assessed | Not assessed | Not assessed | fair | fair | Not assessed | Not assessed | Not assessed |
| Joice et al. (2012) | Perceived usefulness of a stroke workbook-based intervention measure | poor | Not assessed | Not assessed | fair | fair | good | Not assessed | Not assessed | Not assessed |
| Xiao et al. (2014) | Baylor EHR UX survey | poor | Not assessed | Not assessed | fair | Not assessed | Not assessed | Not assessed | Not assessed | Not assessed |
| Weiner et al. (2017)* | Intervention Appropriateness Measure (IAM) | poor | poor | Not assessed | good | poor | Not assessed | Not assessed | Not assessed | Not assessed |
| King et al. (2017) | The Portal Survey on Satisfaction and Impact on Care | poor | Not assessed | Not assessed | poor | Not assessed | Not assessed | Not assessed | Not assessed | Not assessed |
|  | **Adoption (number of instruments=4)** | | | | | | | | | |
| Nydegger et al. (2017) | Strength of Implementation Intentions Scale (SIIS) for condom use | fair | Not assessed | Not assessed | Not assessed | fair | fair | Not assessed | Not assessed | Not assessed |
| Everson et al. (2014) | American Hospital Association IT (AHA-IT) Supplement Survey | fair | Not assessed | Not assessed | poor | fair | Not assessed | Not assessed | fair | Not assessed |
| Malo et al. (2012) | A questionnaire evaluating nurses’ intention to use an electronic medical charting system | poor | poor | Not assessed | fair | poor | Not assessed | poor | Not assessed | Not assessed |
| Kaltenbrunner et al (2017) | Lean in Healthcare Questionnaire (LiHcQ) | excellent | Unable to score** | Not assessed | fair | fair | Not assessed | fair | Not assessed | Not assessed |
|  | **Feasibility (number of instruments=4)** | | | | | | | | | |
| Garcia-Smith et al (2013) | Instrument to test the Clinical Information Systems Success Model (CISSM) | excellent | Not assessed | Not assessed | poor | excellent | good | Not assessed | Not assessed | Not assessed |
| Schnall et al. (2011) | Technology Acceptance Survey | fair | Not assessed | Not assessed | poor | fair | Not assessed | Not assessed | Not assessed | Not assessed |
| Windsor et al. (2013) | The Smoking Cessation and Reduction in Pregnancy Treatment (SCRIPT) Adoption Scale | poor | poor | Not assessed | poor | poor | fair | Not assessed | Not assessed | Not assessed |
| Weiner et al. (2017)* | Feasibility of Intervention Measure (FIM) | poor | poor | Not assessed | good | poor | Not assessed | Not assessed | Not assessed | Not assessed |
|  | **Penetration (number of instruments=4)** | | | | | | | | | |
| Grooten et al. (2019) | The Scaling Integrated Care in Context (SCIROCCO) tool | good | Not assessed | Not assessed | Not assessed | good | good | Not assessed | good | Not assessed |
| Slaghuis et al. (2013) | A measurement instrument for spread of quality improvement in healthcare | fair | Not assessed | Not assessed | poor | fair | Not assessed | Not assessed | Not assessed | Not assessed |
| Flanagan et al. (2007) | The Prevention and Control of Antimicrobial resistance (PACAR) scale | poor | Not assessed | Not assessed | excellent | fair | Not assessed | Not assessed | good | Not assessed |
| Jaana et al. (2005) | A measure of clinical information technology sophistication in hospitals | poor | Not assessed | Not assessed | Not assessed | Not assessed | Not assessed | Not assessed | Not assessed | Not assessed |
|  | **Sustainability (number of instruments=3)** | | | | | | | | | |
| Finch et al. (2018) | Normalisation Measure Development Questionnaire (NoMAD) | fair | Not assessed | Not assessed | excellent | fair | Not assessed | Not assessed | Not assessed | Not assessed |
| Elf et al. (2018) | Normalisation Measure Development Questionnaire (S-NoMAD) - Swedish version | fair | Not assessed | Not assessed | Not assessed | fair | Not assessed | poor | Not assessed | Not assessed |
| Slaghuis et al. (2011) | A measurement instrument for sustainability of work practices in long-term care-Short version | poor | Not assessed | Not assessed | poor | poor | Not assessed | Not assessed | Not assessed | Not assessed |
|  | A measurement instrument for sustainability of work practices in long-term care-Long version | poor | Not assessed | Not assessed | poor | poor | Not assessed | Not assessed | Not assessed | Not assessed |
| Barab et al. (1998) | The Levels of Institutionalization (LoIn) scales | poor | Not assessed | Not assessed | Not assessed | poor | fair | Not assessed | fair | Not assessed |
| *One analysis was reported where the 3 scales were included in the same model.  ** Test-retest reliability procedure not reported therefore unable to score.  NOTE: Underlined rows indicate instruments with multiple versions. | | | | | | | | | | |
