# Supplementary material for: Implementation outcome instruments for use in physical healthcare settings: a systematic review
Source: Implement Sci. 2020 Aug 18;15:66. doi: 10.1186/s13012-020-01027-6 (PMC7433178; doi:10.1186/s13012-020-01027-6)
Supplement: Supplementary file 4 — Additional file 4. ConPsy scores. [file 13012_2020_1027_MOESM4_ESM.docx]

| **Additional file 4. ConPsy scores** | | | | | | | | | | | | | | | | | | |  |  |  |  |  |  |  |  |
| --- | --- | --- | --- | --- | --- | --- | --- | --- | --- | --- | --- | --- | --- | --- | --- | --- | --- | --- | --- | --- | --- | --- | --- | --- | --- | --- |
| **Reference** | **Implementation Outcome**  Name of measurement instrument or instrument description | **Internal consistency** | | **Test-retest or Inter-rater** | | **Validity** | | | | | **Factor analysis** | | | | | | **Penalty*** | **Total score (/22)** |  |  |  |  |  |  |  |  |
|  |  | **IIC/ITC** | **alpha** | **IL** | **FL** | **CV** | **FV** | **CNV** | **DNTV** | **DV** | **AM** | **KC** | **Ve/PA** | **Lo** | **GoF(EFA)** | **GoF(CFA)** |  |  |  |  |  |  |  |  |  |  |
|  | **Acceptability (number of instruments=33)** | | | | | | | | | | | | | | | | | |  |  |  |  |  |  |  |  |
| Shaw et al. (2007) | The Mind the Gap Scale-Adolescent version | 1 | 1 | 0 | 0 | 1 | 1 | 0 | 0 | 1 | 1 | 1 | 0 | 0 | 0 | 0 |  | 7 |  |  |  |  |  |  |  |  |
|  | The Mind the Gap Scale-Parent version | 1 | 1 | 0 | 0 | 1 | 1 | 0 | 0 | 1 | 1 | 1 | 0 | 0 | 0 | 0 |  | 7 |  |  |  |  |  |  |  |  |
| Dow et al. (2013) | The Person-Centred Health Care for Older Adults (PCHCOA) Survey | 0 | 0 | 0 | 0 | 1 | 1 | 0 | 0 | 0 | 1 | 0 | 1 | 1 | 0 | 0 | -2 | **3** |  |  |  |  |  |  |  |  |
| Dykes et al. (2007) | The Impact of Health Information Technology (I-HIT) Scale | 0 | 1 | 0 | 0 | 1 | 1 | 0 | 0 | 0 | 1 | 1 | 1 | 1 | 0 | 0 | -1 | **6** |  |  |  |  |  |  |  |  |
| Brehaut et al. (2010) | Ottawa acceptability of decision rules instrument (OADRI) | 1 | 0 | 0 | 0 | 1 | 0 | 0 | 0 | 1 | 0 | 0 | 0 | 0 | 0 | 0 |  | **3** |  |  |  |  |  |  |  |  |
| Tomotaki et al. (2018) | Evidence-Based Practice Questionnaire (EBPQ‐J) - Japanese version | 0 | 1 | 0 | 0 | 1 | 1 | 0 | 1 | 1 | 0 | 1 | 0 | 0 | 0 | 0 | -2 | **4** |  |  |  |  |  |  |  |  |
| Upton et al. (2006) | Evidence-Based Practice Questionnaire (EBPQ) | 0 | 1 | 0 | 0 | 1 | 1 | 0 | 0 | 1 | 1 | 1 | 1 | 1 | 0 | 0 | -1 | **7** |  |  |  |  |  |  |  |  |
| Bhor et Mason (2006) | A Scale to assess attitudes of health care administrators toward the use of e-mail communication between patients and physicians | 1 | 0 | 0 | 0 | 0 | 1 | 0 | 0 | 1 | 0 | 0 | 0 | 1 | 0 | 0 |  | **4** |  |  |  |  |  |  |  |  |
| Phansalkar et al. (2008) | Instrument for assessing clinicians’ perceptions about use of computerized protocols | 0 | 0 | 0 | 0 | 1 | 1 | 0 | 0 | 0 | 0 | 1 | 1 | 0 | 0 | 0 | -1 | **3** |  |  |  |  |  |  |  |  |
| Oliveira et al. (2011) | CARDIOSATIS-Team scale | 1 | 1 | 0 | 0 | 0 | 0 | 0 | 0 | 1 | 1 | 0 | 1 | 1 | 0 | 0 |  | **6** |  |  |  |  |  |  |  |  |
| Wu et al. (2008) | Healthcare professionals' intention to use an adverse event reporting system | 0 | 1 | 0 | 0 | 1 | 0 | 0 | 0 | 0 | 0 | 0 | 0 | 0 | 0 | 4 |  | **6** |  |  |  |  |  |  |  |  |
| Melas et al. (2012) | The Evidence-Based Practice Attitude Scale (EBPAS)-Greek version | 0 | 0 | 0 | 0 | 0 | 0 | 0 | 0 | 1 | 0 | 1 | 1 | 0 | 0 | 2 |  | **5** |  |  |  |  |  |  |  |  |
| Brouwers et al. (2004) | Practitioner Feedback Questionnaire | 0 | 1 | 0 | 0 | 1 | 1 | 0 | 0 | 0 | 0 | 0 | 1 | 1 | 0 | 0 | -1 | **4** |  |  |  |  |  |  |  |  |
| Baker et al. (2016) | The Attitudes Related to Trauma-Informed Care (ARTIC-45) | 0 | 1 | 0 | 0 | 1 | 0 | 0 | 0 | 0 | 0 | 0 | 0 | 0 | 0 | 2 |  | **4** |  |  |  |  |  |  |  |  |
|  | The Attitudes Related to Trauma-Informed Care (ARTIC-35) Scale | 0 | 1 | 0 | 0 | 1 | 0 | 0 | 0 | 0 | 0 | 0 | 0 | 0 | 0 | 2 |  | **4** |  |  |  |  |  |  |  |  |
|  | The Attitudes Related to Trauma-Informed Care (ARTIC-10) Scale-Short version | 0 | 1 | 0 | 0 | 1 | 0 | 0 | 0 | 0 | 0 | 0 | 0 | 0 | 0 | 0 |  | **2** |  |  |  |  |  |  |  |  |
| Vanneste et al. (2013) | A survey measuring acceptance of BelRAI, a web-based system enabling person-centred recording and data sharing across care settings. | 0 | 2 | 0 | 0 | 0 | 0 | 0 | 0 | 0 | 0 | 0 | 0 | 0 | 0 | 0 |  | **2** |  |  |  |  |  |  |  |  |
| Bakas et al. (2009) | A rating form measuring the satisfaction of the Telephone Assessment and Skill-Building Kit (TASK) intervention. | 0 | 1 | 0 | 1 | 1 | 1 | 0 | 0 | 0 | 0 | 0 | 0 | 0 | 0 | 0 | -1 | **3** |  |  |  |  |  |  |  |  |
| McConnell et al. (2012) | Diffusion of Innovation in Long-Term Care (DOI-LTC) measurement battery-version for certified nursing assistants | 0 | 0 | 0 | 0 | 1 | 1 | 0 | 0 | 0 | 0 | 0 | 0 | 0 | 0 | 0 |  | **2** |  |  |  |  |  |  |  |  |
|  | Diffusion of Innovation in Long-Term Care (DOI-LTC) measurement battery-version for licensed nurses | 0 | 0 | 0 | 0 | 1 | 1 | 0 | 0 | 0 | 0 | 0 | 0 | 0 | 0 | 0 |  | **2** |  |  |  |  |  |  |  |  |
| Atkinson (2007) | A Questionnaire to Measure Perceived Attributes of eHealth Innovations | 0 | 1 | 0 | 0 | 0 | 0 | 0 | 0 | 0 | 0 | 1 | 1 | 1 | 0 | 0 |  | **4** |  |  |  |  |  |  |  |  |
| Gagnon et al. (2012) | A questionnaire based on the Technology Acceptance Model (TAM) | 0 | 0 | 0 | 0 | 1 | 1 | 0 | 0 | 1 | 0 | 0 | 0 | 0 | 0 | 0 |  | **3** |  |  |  |  |  |  |  |  |
| Ferrando et al. (2010) | A questionnaire to measure convenience and satisfaction with a new internet-based tool for oral anticoagulation therapy telecontrol | 0 | 0 | 0 | 0 | 1 | 1 | 0 | 0 | 0 | 0 | 0 | 0 | 0 | 0 | 0 |  | **2** |  |  |  |  |  |  |  |  |
| Wilkinson et al. (2018) | A survey measuring attitudes towards biomedical HIV prevention | 0 | 0 | 0 | 0 | 0 | 0 | 0 | 0 | 0 | 0 | 1 | 1 | 1 | 0 | 0 | -1 | **2** |  |  |  |  |  |  |  |  |
| Adu et al. (1999) | A questionnaire measuring pharmacists and physician’s attitudes to antibiotic policies | 0 | 1 | 0 | 0 | 0 | 0 | 0 | 0 | 0 | 0 | 0 | 0 | 1 | 0 | 0 |  | **2** |  |  |  |  |  |  |  |  |
| Abetz et al. (2005) | Cancer Therapy Satisfaction Questionnaire (CTSQ) | 0 | 0 | 0 | 0 | 1 | 1 | 0 | 0 | 0 | 0 | 0 | 0 | 0 | 0 | 0 |  | **2** |  |  |  |  |  |  |  |  |
| Blumenthal et al. (2018) | Physiotherapy Mobile Acceptance Questionnaire (PTMAQ) | 1 | 1 | 0 | 0 | 1 | 1 | 0 | 0 | 1 | 1 | 1 | 1 | 1 | 0 | 0 |  | **9** |  |  |  |  |  |  |  |  |
| Weiner et al. (2017)* | Acceptability of Intervention Measure (AIM) | 0 | 2 | 0 | 0 | 1 | 1 | 0 | 0 | 1 | 0 | 0 | 0 | 0 | 0 | 3 |  | **8** |  |  |  |  |  |  |  |  |
| Unni et al. (2016) | A survey measuring satisfaction with Electronic health records | 0 | 2 | 0 | 0 | 1 | 0 | 0 | 0 | 0 | 0 | 1 | 1 | 1 | 0 | 0 |  | **6** |  |  |  |  |  |  |  |  |
| Aggelidis et al. (2012) | End user computing satisfaction (EUCS) survey | 0 | 2 | 0 | 0 | 0 | 0 | 1 | 0 | 0 | 1 | 0 | 1 | 1 | 0 | 0 |  | **6** |  |  |  |  |  |  |  |  |
| El-Den et al. (2018) | Perinatal Depression (PND) Attitudes and Screening Acceptability Questionnaire (PASAQ) | 0 | 0 | 0 | 0 | 1 | 1 | 0 | 0 | 0 | 1 | 1 | 1 | 1 | 0 | 0 | -1 | **5** |  |  |  |  |  |  |  |  |
| Kramer et al. (2014) | A generic questionnaire to detect physicians’ willingness to implement complex medical interventions | 0 | 1 | 0 | 0 | 1 | 1 | 0 | 0 | 0 | 0 | 0 | 0 | 0 | 0 | 2 |  | **5** |  |  |  |  |  |  |  |  |
| Frandes et al. (2017) | An instrument assessing mobile technology acceptability in diabetes self-management | 0 | 0 | 0 | 0 | 1 | 1 | 0 | 0 | 0 | 1 | 0 | 1 | 1 | 0 | 0 | -1 | **4** |  |  |  |  |  |  |  |  |
| Rasoulzadeh et al. (2017) | A questionnaire measuring acceptance of creating a nurses' health monitoring system | 0 | 1 | 0 | 0 | 1 | 1 | 0 | 0 | 0 | 0 | 0 | 0 | 0 | 0 | 0 |  | **3** |  |  |  |  |  |  |  |  |
| Sockolow et al. (2011) | Electronic Health Record Nurse Satisfaction (EHRNS) survey | 0 | 0 | 0 | 0 | 1 | 1 | 0 | 0 | 1 | 0 | 0 | 0 | 0 | 0 | 0 |  | **3** |  |  |  |  |  |  |  |  |
| Johnston et al. (2002) | A questionnaire assessing physicians' attitudes towards the computerization of clinical practice | 0 | 0 | 0 | 0 | 0 | 0 | 0 | 0 | 1 | 0 | 1 | 0 | 1 | 0 | 0 | -1 | **2** |  |  |  |  |  |  |  |  |
| Bernhardsson et al. (2013) | Evidence-Based Practice (EBP) questionnaire | 0 | 0 | 0 | 0 | 1 | 1 | 0 | 0 | 0 | 0 | 0 | 0 | 0 | 0 | 0 |  | **2** |  |  |  |  |  |  |  |  |
| Yildiz et al. (2018) | Evidence-Based Practice Attitude Scale (EBPAS-50) - Turkish version | 0 | 0 | 0 | 0 | 0 | 0 | 0 | 0 | 0 | 1 | 0 | 0 | 0 | 0 | 0 | -1 | **0** |  |  |  |  |  |  |  |  |
| Bevier et al. (2014) | Questionnaire of three scoring items for current treatment satisfaction and factors of both clinical trial participation motivations and technology acceptance model | 0 | 0 | 0 | 0 | 0 | 0 | 0 | 0 | 0 | 0 | 0 | 0 | 0 | 0 | 0 |  | **0** |  |  |  |  |  |  |  |  |
| Silver Wolf et al. (2014) | Evidence-Based Practice Attitude Scale (EBPAS) | 0 | 0 | 0 | 0 | 0 | 0 | 0 | 0 | 1 | 0 | 0 | 0 | 0 | 0 | 4 |  | **5** |  |  |  |  |  |  |  |  |
| Steed et al. (2008) | Acceptability of Continuous Glucose Monitoring Devices (ACGMD) questionnaire | 0 | 0 | 0 | 0 | 1 | 1 | 0 | 0 | 0 | 0 | 0 | 0 | 0 | 0 | 0 |  | **2** |  |  |  |  |  |  |  |  |
|  | **Appropriateness (number of instruments=7)** | | | | | | | | | | | | | | | | | |  |  |  |  |  |  |  |  |
| Diego et al. (2016) | A questionnaire to measure the attitude of anesthesiologists and residents regarding the use of the checklist in the perioperative period | 0 | 0 | 0 | 0 | 0 | 0 | 0 | 0 | 1 | 0 | 0 | 1 | 0 | 0 | 0 |  | **2** |  |  |  |  |  |  |  |  |
| Park et al. (2016) | A questionnaire measuring motivational factors for using wearable healthcare devices | 0 | 2 | 0 | 0 | 1 | 1 | 0 | 0 | 0 | 0 | 0 | 0 | 0 | 0 | 4 |  | **8** |  |  |  |  |  |  |  |  |
| Razmak et al. (2018) | A Techno-humanist model for e-health adoption of innovative technology | 0 | 1 | 0 | 0 | 0 | 0 | 0 | 0 | 1 | 0 | 0 | 0 | 0 | 0 | 0 | -1 | **1** |  |  |  |  |  |  |  |  |
| Joice et al. (2012) | Perceived usefulness of a stroke workbook-based intervention measure | 0 | 1 | 0 | 0 | 1 | 1 | 0 | 0 | 1 | 0 | 1 | 0 | 1 | 0 | 0 |  | **6** |  |  |  |  |  |  |  |  |
| Xiao et al. (2014) | Baylor EHR UX survey | 0 | 1 | 0 | 0 | 1 | 1 | 0 | 0 | 0 | 0 | 0 | 0 | 0 | 0 | 0 |  | **3** |  |  |  |  |  |  |  |  |
| Weiner et al. (2017)* | Intervention Appropriateness Measure (IAM) | 0 | 2 | 0 | 0 | 1 | 1 | 0 | 0 | 1 | 0 | 0 | 0 | 0 | 0 | 3 |  | **8** |  |  |  |  |  |  |  |  |
| King et al. (2017) | The Portal Survey on Satisfaction and Impact on Care | 0 | 0 | 0 | 0 | 0 | 0 | 1 | 1 | 0 | 1 | 0 | 1 | 1 | 0 | 0 |  | **5** |  |  |  |  |  |  |  |  |
|  | **Adoption** **(number of instruments=4)** | | | | | | | | | | | | | | | | | |  |  |  |  |  |  |  |  |
| Nydegger et al. (2017) | Strength of Implementation Intentions Scale (SIIS) for condom use | 0 | 0 | 0 | 0 | 0 | 0 | 0 | 0 | 0 | 0 | 0 | 0 | 0 | 0 | 4 |  | **4** |  |  |  |  |  |  |  |  |
| Everson et al. (2014) | American Hospital Association IT (AHA-IT) Supplement Survey | 1 | 1 | 0 | 0 | 1 | 1 | 0 | 0 | 0 | 0 | 1 | 1 | 0 | 0 | 0 |  | **6** |  |  |  |  |  |  |  |  |
| Malo et al. (2012) | A questionnaire evaluating nurses’ intention to use an electronic medical charting system | 0 | 1 | 0 | 0 | 1 | 1 | 0 | 0 | 0 | 0 | 0 | 0 | 0 | 0 | 0 |  | **3** |  |  |  |  |  |  |  |  |
| Kaltenbrunner et al (2017) | Lean in Healthcare Questionnaire (LiHcQ) | 0 | 0 | 0 | 1 | 0 | 1 | 0 | 0 | 1 | 0 | 0 | 0 | 0 | 0 | 4 |  | **7** |  |  |  |  |  |  |  |  |
|  | **Feasibility (number of instruments=4)** | | | | | | | | | | | | | | | | | |  |  |  |  |  |  |  |  |
| Garcia-Smith et al (2013) | Instrument to test the Clinical Information Systems Success Model (CISSM) | 0 | 1 | 0 | 0 | 0 | 0 | 0 | 0 | 0 | 0 | 0 | 0 | 1 | 0 | 0 |  | **2** |  |  |  |  |  |  |  |  |
| Schnall et al. (2011) | Technology Acceptance Survey | 0 | 1 | 0 | 0 | 0 | 0 | 0 | 0 | 1 | 0 | 0 | 1 | 1 | 0 | 0 | -1 | **3** |  |  |  |  |  |  |  |  |
| Windsor et al. (2013) | The Smoking Cessation and Reduction in Pregnancy Treatment (SCRIPT) Adoption Scale | 0 | 1 | 0 | 0 | 1 | 0 | 0 | 0 | 0 | 0 | 0 | 0 | 0 | 0 | 2 |  | **4** |  |  |  |  |  |  |  |  |
| Weiner et al. (2017)* | Feasibility of Intervention Measure (FIM) | 0 | 2 | 0 | 0 | 1 | 1 | 0 | 0 | 1 | 0 | 0 | 0 | 0 | 0 | 3 |  | **8** |  |  |  |  |  |  |  |  |
|  | **Penetration (number of instruments=4)** | | | | | | | | | | | | | | | | | |  |  |  |  |  |  |  |  |
| Grooten et al. (2019) | The Scaling Integrated Care in Context (SCIROCCO) tool | 0 | 0 | 0 | 0 | 0 | 0 | 1 | 1 | 0 | 1 | 0 | 1 | 1 | 0 | 0 |  | **5** |  |  |  |  |  |  |  |  |
| Slaghuis et al. (2013) | A measurement instrument for spread of quality improvement in healthcare | 0 | 1 | 0 | 0 | 1 | 0 | 0 | 0 | 0 | 0 | 0 | 0 | 0 | 0 | 2 |  | **4** |  |  |  |  |  |  |  |  |
| Flanagan et al. (2007) | The Prevention and Control of Antimicrobial resistance (PACAR) scale | 0 | 0 | 0 | 0 | 1 | 1 | 0 | 0 | 0 | 0 | 0 | 1 | 1 | 0 | 2 |  | **6** |  |  |  |  |  |  |  |  |
| Jaana et al. (2005) | A measure of clinical information technology sophistication in hospitals | 1 | 1 | 0 | 0 | 0 | 0 | 0 | 0 | 1 | 0 | 0 | 0 | 0 | 0 | 0 |  | **3** |  |  |  |  |  |  |  |  |
|  | **Sustainability (number of instruments=3)** | | | | | | | | | | | | | | | | | |  |  |  |  |  |  |  |  |
| Finch et al. (2018) | Normalisation Measure Development Questionnaire (NoMAD) | 0 | 1 | 0 | 0 | 1 | 1 | 0 | 0 | 0 | 0 | 0 | 0 | 0 | 0 | 4 |  | **7** |  |  |  |  |  |  |  |  |
| Elf et al. (2018) | Normalisation Measure Development Questionnaire (S-NoMAD) - Swedish version | 0 | 1 | 0 | 0 | 1 | 1 | 0 | 0 | 0 | 0 | 0 | 0 | 0 | 0 | 4 |  | **7** |  |  |  |  |  |  |  |  |
| Slaghuis et al. (2011) | A measurement instrument for sustainability of work practices in long-term care-Short version | 0 | 1 | 0 | 0 | 1 | 0 | 0 | 0 | 1 | 0 | 0 | 0 | 0 | 0 | 4 |  | **7** |  |  |  |  |  |  |  |  |
|  | A measurement instrument for sustainability of work practices in long-term care-Long version | 0 | 1 | 0 | 0 | 1 | 0 | 0 | 0 | 1 | 0 | 0 | 0 | 0 | 0 | 3 |  | **6** |  |  |  |  |  |  |  |  |
| Barab et al. (1998) | The Levels of Institutionalization (LoIn) scales | 0 | 0 | 0 | 0 | 0 | 0 | 0 | 0 | 1 | 0 | 0 | 0 | 0 | 0 | 3 |  | **4** |  |  |  |  |  |  |  |  |
| IIC/ITC: Item internal consistency / Item total correlation; IL: Item level; FL: Factor level; CV: Content validity; FV: Face validity; CNV: Convergent validity; DNTV: Discriminant validity; DV: discriminative validity; AM: Adequacy measures; KC: Kaiser criterion; Ve/PA: Variance explained/ Parallel analysis; Lo: Main loadings; GoF(EFA): EFA goodness of fit; GoF(CFA): CFA goodness of fit  *One analysis was reported where the 3 scales were included in the same model.  NOTE: Underlined rows indicate instruments with multiple versions. | | | | | | | | | | | | | | | | | | | |  |  |  |  |  |  |  |
